# MSCA HOPE - Physical education in hospital schools: A protocol paper for systematic and umbrella reviews with policy framework analysis

**DOI:** 10.12688/openreseurope.23993.1

**Published:** 2026-06-01

**Authors:** Simone Ciaccioni, Caterina Pesce, Valentin Benzing, Mirko Schmidt, Paula Magalhães, Pedro Rosário, Luciana Pedrosa Leal, Cleide Maria Pontes, Livia Oddi, Filippo Betello, Slaviša Bradić, Laura Capranica

**Affiliations:** 1 Department of Movement, Human and Health Sciences, University of Rome “Foro Italico”, Rome, Italy, 00135, Italy; 2 Department of Kinesiology, Michigan State University, East Lansing, MI, 48824, USA; 3 Department of Sport Pedagogy, University of Bern, Bern, 3012, Switzerland; 4 School of Psychology, University of Minho, Braga, 4710-057, Portugal; 5 Department of Nursing, Federal University of Pernambuco, Recife, PE, 50670-420, Brazil; 6 Department of Information Engineering, Control and Management, Sapienza University of Rome, Rome, 00185, Italy; 7 Department of Computer, Control and Management Engineering, Sapienza University of Rome, Rome, 00185, Italy; 8 International Judo Federation Academy Foundation, Malta, XBX 1421, Malta

**Keywords:** sport science, hospitalised youth, systematic literature review, barriers and facilitators, interprofessional collaboration, physical activity programs, health–education integration, evidence-informed policy.

## Abstract

Hospital school programs ensure educational continuity for children and adolescents during hospitalization, yet the inclusion of physical education (PE) in these settings remains inconsistent and lacks unified standards. PE is widely recognized to confer significant physical, psychosocial, and educational benefits, but it is often omitted from hospital-based education support due to unique contextual constraints. This study protocol presents a comprehensive approach to synthesize evidence and inform practice on PE in hospital schools, encompassing a two-tiered review: Tier 1 is an umbrella review of existing systematic reviews on physical activity interventions for hospitalised youth, and Tier 2 is a systematic review of primary studies evaluating PE delivered in hospital school settings. In parallel, a policy framework analysis will be performed to map national and international regulations governing PE in hospital schools. Multiple databases (including MEDLINE, CINAHL, Embase, PsycINFO, Scopus, and SPORTDiscus) will be searched without language or date restrictions, and supplement with reference checks and expert consultations. Study selection and data extraction will be carried out independently by two reviewers. The quality of included studies and policy documents will be appraised using validated tools such as Cochrane RoB2, ROBINS-I, AMSTAR-2 and AGREE-II. Outcomes of interest include physical, cognitive, psychosocial, clinical, and implementation measures. A narrative synthesis is planned, with meta-analyses conducted if sufficient homogeneous data exist; qualitative findings will be integrated via thematic synthesis. The Grading of Recommendations Assessment, Development and Evaluation (GRADE) approach will be applied to assess certainty of evidence. This review will provide an evidence base and policy analysis to support the development of guidelines and best practices for implementing PE in hospital schools. PROSPERO ID registration: CRD420251156215.

## Plain Language summary

Children and adolescents who spend time in hospital often continue their education through hospital school programmes. These programmes help them stay connected with learning, teachers, and classmates. However, physical education is not always included.

This is important because movement, play, exercise, and physical education can support children’s physical health, emotional wellbeing, social participation, and learning. In hospital settings, however, physical education can be difficult to organise. Children may have different health conditions, energy levels, treatment schedules, and safety needs. Hospitals and schools may also follow different rules, and there are no shared standards to guide practice.

This study will bring together the best available knowledge on physical education and physical activity for children and adolescents in hospital settings. The researchers will review previous research studies and examine existing policies and guidelines from different countries and organisations. They will look at what has been studied, what seems helpful, what evidence is still missing, and what rules or recommendations already exist.

The study will consider outcomes related to physical health, mental and social wellbeing, learning, clinical conditions, and practical delivery. It will also assess how reliable the available evidence is.

The results will help build a clearer evidence base for future guidance on physical education in hospital schools. The long-term aim is to support teachers, healthcare professionals, schools, families, and policymakers in offering safe, meaningful, and inclusive physical education opportunities for children and adolescents during hospitalisation.

This review protocol is registered in PROSPERO: CRD420251156215.

## 1 Introduction

Hospital schools provide educational services to children and adolescents during hospitalization, helping to maintain academic progress and a sense of normality despite illness ( Issaka and Hopkins, 2015; Jiliberto and Zarate Alva, 2024). However, delivering a full breadth of curricula in hospital settings is challenging, and physical education (PE) is among the most frequently overlooked components. In many European countries, hospital school programs operate with considerable variability and a lack of standardized guidelines, leading to inconsistencies in practice ( Jiliberto and Zarate Alva, 2024). Hospital teachers frequently face resource and training constraints, as hospital schools often lack sufficient staff, time, and equipment dedicated to PE ( Catenazzo, 2019; Cistulli et al., 2025). As a result, hospitalised youth may miss out on structured opportunities for physical activity and play that are routinely available in regular school environments. Recent evidence suggests that children undergoing extended hospitalizations (e.g., stays exceeding 7–14 days) may miss multiple weeks of regular PE lessons, with some estimates indicating that over 50% of paediatric inpatients experience moderate-to-long-term absences from school ( Davies et al., 2014; Gold et al., 2016; Ludgério et al., 2023). Moreover, qualitative reports highlight that children perceive the lack of physical activity as contributing to boredom, emotional distress, and a sense of social exclusion ( Issaka and Hopkins, 2015; Martins et al., 2021).

Physical activity and PE are critical for the physical health, mental well-being, and social development of youth ( Biddle et al., 2019; World Health Organization, 2022). Moreover, they can improve physical fitness, mood, and cognitive function, and help children retain a sense of normal childhood experiences even while in clinical care ( Metzler et al., 2013; Bull et al., 2020). PE is widely acknowledged to yield important educational, social, and health benefits for students; yet, it is not often included in hospital-based teaching programs ( Issaka and Hopkins, 2015; Theobald et al., 2025). Barriers such as medical fragility of students, safety concerns, limited space or equipment, and the use of generalist teachers (rather than PE specialists) in hospitals all contribute to the omission of PE from hospital schooling ( Pagnani et al., 2004; Issaka and Hopkins, 2015). Nonetheless, emerging pilot initiatives suggest that adapted PE programs can be feasible in hospitals and positively received by students and staff ( Issaka and Hopkins, 2015; Cistulli et al., 2025). This underscores the need to systematically examine what has been investigated in this context and with what outcomes.

To date, there has been no comprehensive review focusing on PE in hospital schools. Some literature has explored broader physical activity or therapeutic exercise interventions for children in hospitals, but these studies are scattered across disciplines (e.g., education, paediatric rehabilitation, psychology) and have not been synthesized to inform educational practice ( Dixon, 2020; Ludgério et al., 2023; Theobald et al., 2025). Moreover, beyond the existing research literature, limited attention has been given to the policies and regulations that shape hospital school curricula, particularly regarding whether and how PE should be provided ( Commission et al., 2013; Catenazzo, 2019). International frameworks (e.g., United Nations, World Health Organisation, national education and health ministries) affirm the right to inclusive and equitable education for hospitalised children ( McLennan and Thompson, 2015; UNESCO, 2015; Bull et al., 2020), but specific guidance on integrating physical activity into hospital education is limited ( Parliament and Union, 2016). Furthermore, European initiatives such as the Youth Wiki, an online platform documenting national youth policies across Europe, highlight the importance of understanding the policy context. National governance structures, funding priorities, and levels of intersectoral coordination shape the resources and institutional support available to hospital schools, potentially enabling or constraining the implementation of PE ( Commission et al., 2025).

In response to these gaps, a multi-component review has been designed to consolidate evidence and policy information on PE in hospital schools. The specific objectives are to: (i) identify and describe the characteristics, content, and implementation modalities of PE interventions in hospital school settings; (ii) evaluate the feasibility and effectiveness of such interventions, including their physical, psychosocial, and educational outcomes; (iii) synthesize higher-level evidence from existing systematic reviews (umbrella review) on physical activity interventions in paediatric hospital contexts, to discern transferable lessons for hospital school PE; (iv) map and analyse national and international policy and regulatory frameworks for the delivery of PE and physical activity in hospital schools, including provisions for teacher education and preparation in hospital-based PE; (v) integrate empirical findings with policy analysis to generate evidence-informed recommendations for educators, healthcare professionals, and policymakers on promoting and standardizing PE in hospital school programs.

Thus, developed within the framework of the MSCA Global Postdoctoral Fellowship project “Hospital schools’ guidelines On Physical Education” (HOPE; https://cordis.europa.eu/project/id/101206417), this research aims to contribute to advancing evidence-based practices and inclusive educational policies. Aligned with the EU’s goals for promoting quality, equity, and innovation in education ( European Union, 2008; European Commission, 2023), the findings will underpin a coordinated, international, and interdisciplinary strategy to ensure that hospitalised children and adolescents benefit from high-quality PE. These efforts are intended to enhance not only their physical and psychosocial well-being, but also to support educational continuity and long-term development in alignment with the principles of child-centred care and inclusive schooling ( McLennan and Thompson, 2015).

## 2 Methods and analysis

### 2.1 Review structure

The present protocol was prospectively registered in the International Prospective Register of Systematic Reviews (PROSPERO) on 04 December 2025 (registration number: CRD420251156215). The preparation and reporting of this protocol manuscript were conducted in accordance with the Preferred Reporting Items for Systematic Review and Meta-Analysis Protocols (PRISMA-P; see Extended Data: ( Ciaccioni et al., 2026)) guidelines ( Shamseer et al., 2015). A combined systematic review, umbrella review, and policy analysis design will be employed. The overall methodology is structured in two tiers with a parallel policy component:

• Tier 1: Umbrella Review of Systematic Reviews. An umbrella review will be performed to compile findings from existing systematic reviews and meta-analyses that address physical activity interventions in hospitalised children and adolescents (not limited to school-provided programs). This higher-level synthesis will provide context and broader evidence, for example on exercise programs led by healthcare providers in hospital settings, which could inform practices in hospital schools. Including an umbrella review allows evaluating the consistency of evidence across multiple reviews and to identify generalizable insights or areas of consensus regarding physical activity benefits, risks, and implementation in paediatric hospital contexts ( Biddle et al., 2019; Ludgério et al., 2023).
• Tier 2: Systematic Review of Primary Studies. This component will identify and synthesize quantitative and qualitative primary research studies that evaluated PE in hospital school settings. Eligible study designs span randomized controlled trials (RCTs), quasi-experimental studies, non-randomized and observational studies (e.g., cohort or cross-sectional studies), qualitative research (e.g., interviews, focus groups), mixed-methods studies, and informative case reports ( Tenenbaum and Driscoll, 2005). This breadth is necessary given the expected limited number of RCTs in this recently developing field; studies may be single-arm program evaluations or purely qualitative descriptions.
• Policy Framework Analysis. In addition to the empirical literature reviews, a narrative policy and regulatory analysis will be conducted. This will involve identifying relevant policy documents, guidelines, and regulations from governmental and international bodies (e.g., education or health ministries, hospital school governing bodies, UNESCO and WHO publications, and relevant legislation) that pertain to PE or physical activity for hospitalised students ( McLennan and Thompson, 2015; World Health Organization, 2020). To locate relevant policy documents, grey literature sources, and institutional repositories will be searched, and experts consulted (including through the HOPE network of hospital educators). The analysis will map the landscape of policies across different countries and levels (regional, national, international) and critically examine how these frameworks address (or fail to address) the inclusion of physical activity in hospital education. A summary will be provided for the scope and enforcement of existing regulations, cross-national variations in policy, and the degree of alignment (or misalignment) between policy directives and the empirical evidence base ( Bowen, 2009; Browne et al., 2019).

**Figure 1.**
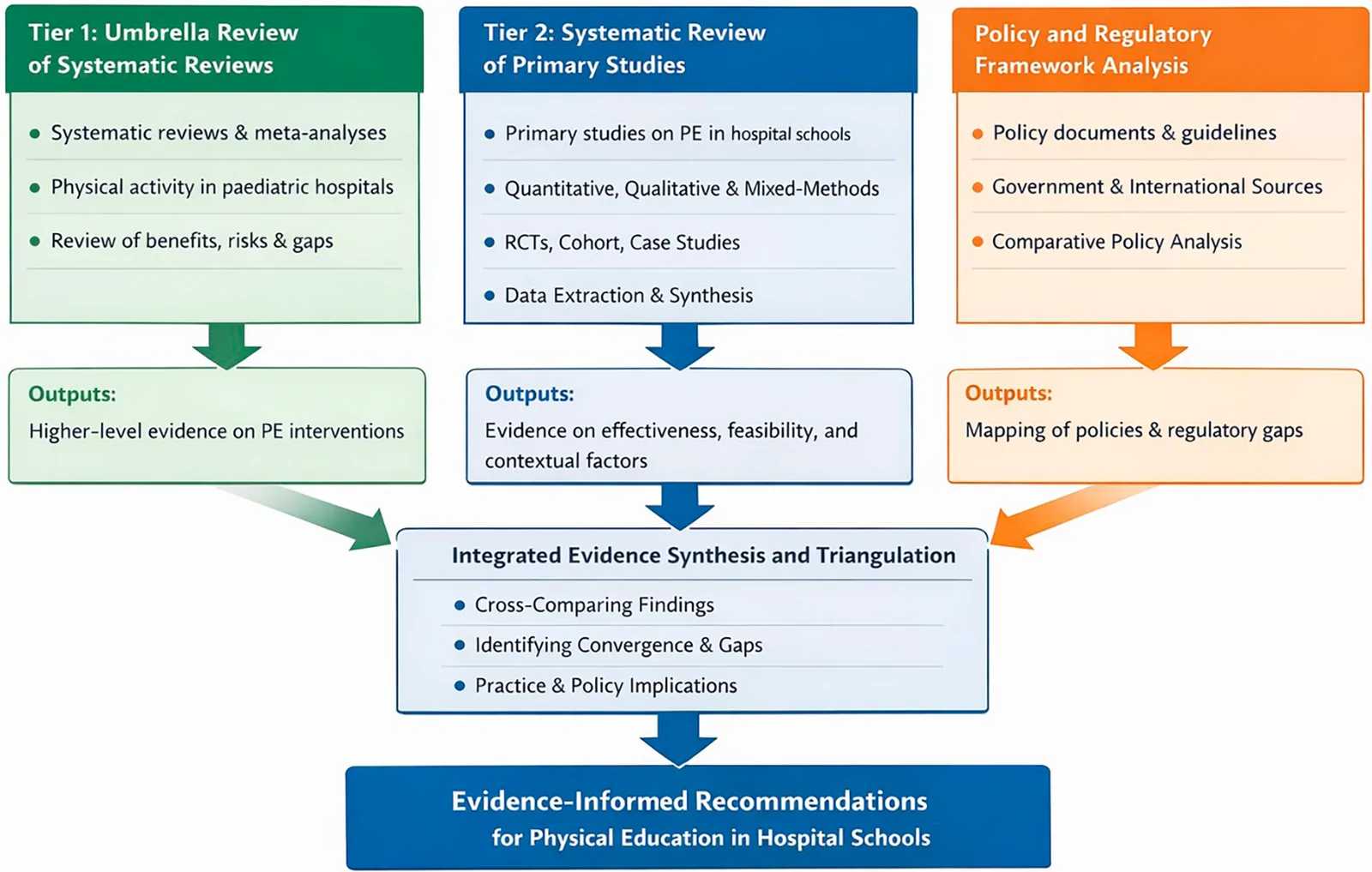
Overview of the concurrent multi-tier design and evidence integration. Note: PE = Physical education; RCT = Randomised controlled trial.

This multi-faceted design ( Figure 1) enables both depth and breadth in the present review. Tier 1 provides a focused examination of hospital school PE interventions, Tier 2 offers a broad overview of related evidence in hospitals, and the policy analysis connects these findings to real-world governance and practice guidelines.

### 2.2 Eligibility criteria

Inclusion and exclusion criteria were defined according to the PICOS (Population, Intervention, Comparator, Outcome, Study design) framework ( Brown et al., 2006), tailored to each tier of the review as appropriate.

**Population**: The review will consider studies involving children and adolescents aged approximately 5–18 years (school-age youth from primary through secondary level) who received education in a hospital-based school or educational program during hospitalization ( Issaka and Hopkins, 2015). This includes any paediatric patients enrolled in formal hospital schools (accredited on-site schools within hospitals) or informal educational services provided by hospitals, regardless of the students’ medical diagnoses or conditions. Studies will be included across all health conditions (e.g., acute illnesses, chronic diseases, mental or physical health conditions, injuries) and from any geographic location or healthcare system ( Reid, 2010). The key criterion is that the education/intervention occurs during an in-patient hospital stay or as part of a hospital-associated educational program. The studies focusing on children receiving education exclusively at home or in outpatient settings (such as home tuition or clinic-based schooling) will be excluded unless those interventions are explicitly part of a coordinated hospital school program or a transition program from hospital to home ( Catenazzo, 2019).

**Interventions**: Any intervention or program will be included if described as a form of PE, physical activity, exercise, or play-based movement activity delivered within the context of a hospital school or during a hospital stay as part of the child’s educational and therapeutic activities ( Cistulli et al., 2025). This encompasses a wide variety of possible interventions, for example: (i) curriculum-based PE lessons provided by hospital school teachers, such as adapted PE classes or movement activities aligned with the standard school curriculum but modified for the hospital environment ( Issaka and Hopkins, 2015); (ii) therapeutic exercise or rehabilitation programs integrated into schooling, for instance exercise sessions led collaboratively by physiotherapists or other healthcare staff in coordination with educators, such as structured physical therapy activities scheduled as part of the school day ( Ludgério et al., 2023); (iii) play-based or recreational physical activities delivered by teachers, play specialists, or therapists, including group games, dance, martial arts, yoga, sports, or other forms of active play. These activities may take place in hospital playrooms or classrooms, or in home settings when formally integrated within hospital-based interventions, and are designed to promote enjoyable movement experiences and facilitate social interaction among children ( Benzing et al., 2020; Marusak et al., 2020). Interventions of any intensity, frequency, or duration, from one-off sessions to long-term programs will be included. Considering that hospital-based programs might use various terminologies (e.g. “therapeutic play” or “exercise therapy”) even if the core activity is physical and educational in nature, an inclusive approach will be taken without a strict threshold for what “counts” as PE, if the authors describe the activity as PE, exercise, physical activity, or similar, and it occurs in the hospital educational setting. If available, information on who delivers the interventions (e.g., academic background, regulations) will be extracted.

To integrate evidence from therapeutic and health-focused programs that may inform best practices in hospital school contexts, at the umbrella review level (Tier 1) the scope will be broadened to include any hospital-based physical activity interventions for children, even if they are not explicitly labelled as educational or part of a school curriculum. For instance, a systematic review of exercise interventions for hospitalised children (led by healthcare providers for rehabilitation or health outcomes) would be included, as those findings could yield transferable insights for hospital school PE ( Santos et al., 2020). In Tier 2, interventions lacking an educational or school-related component within the hospital setting will be excluded. Specifically, studies focusing exclusively on clinical physical therapy or rehabilitation delivered without an explicit educational objective or linkage to a hospital school program will not be eligible for inclusion in the systematic review. For instance, inpatient exercise regimens implemented solely as medical therapy without an educational context will not meet the Tier 2 inclusion criteria, although they may be captured within Tier 1 if discussed in an eligible review. This exclusion criterion is applied to preserve a clear conceptual focus on interventions aligned with the educational mission of hospital schools. In addition, interventions conducted entirely outside the hospital school timeframe or setting, such as post-discharge community sport activities not embedded within a formal educational transition plan, will be excluded in accordance with the defined population and context criteria.

**Comparator**: Given the anticipated heterogeneity of study designs, the presence of a comparator or control group will not be required for inclusion. Many eligible studies, particularly qualitative investigations and single-arm programme evaluations, are expected to lack a control condition. When a comparator (e.g., usual care, non-PE activities, or pre–post comparisons) is included, this information will be extracted and considered in the analysis, but its absence will not constitute an exclusion criterion. This approach reflects the exploratory nature of the field and aims to avoid the exclusion of relevant descriptive evidence. Where applicable, the implications of including uncontrolled studies will be addressed during risk-of-bias and study quality assessment and interpretation of the overall evidence (see section: 2.6 Risk of Bias and Quality Assessment).

**Outcomes**: A broad range of domains related to the impact or implementation of PE/physical activity programs in the hospital school context, including the following spheres: (i) body (e.g., measures of physical fitness, motor skills, functional mobility, strength, endurance; rehabilitation progress indicators; pain levels; other clinical physical health metrics); (ii) cognition and education (e.g., attention and concentration, cognitive function tests, academic performance or progress, school engagement, achievement of educational milestones despite illness, and indicators of learning continuity); (iii) psychosocial outcomes (e.g., mental and emotional well-being such as mood, anxiety, depression symptoms, self-esteem, enjoyment and motivation, social interaction and inclusion with peers or teachers, quality of life related to psychosocial health, resilience, and the child’s adjustment to hospitalization); (iv) health and clinical outcomes (e.g., health-related quality of life, adherence to treatment or rehabilitation protocols, clinical recovery rates or functional recovery, length of hospital stay if influenced by the intervention, and patient satisfaction with care or with the hospital school experience); (v) implementation and process measures (e.g., feasibility of the program, delivery as intention, recruitment/retention rates, participation and engagement levels of students, adherence to the activity sessions, any safety incidents or adverse events, and qualitative feedback from children, parents, or staff about the program). Descriptive information will be also noted about how the intervention was delivered (setting, schedule, class size), who delivered it (teacher vs. therapist, or a team), and any theoretical frameworks or guidelines referenced in program design ( Pesce et al., 2021; Stodden et al., 2023). No primary outcome is predefined due to the exploratory nature of the protocol: as a study is eligible if it reports at least one relevant outcome in any domain, systematic reviews focusing on specific outcome domains are also eligible.

**Study Designs**: For Tier 1, the included study designs are qualitative reviews and quantitative meta-analyses. Any review that used systematic search and inclusion methods to synthesize studies on hospital-based physical activity will be included. Purely theoretical papers, editorials, opinion pieces, and commentaries that do not present new data will be excluded. Only full studies with outcome data will be synthesized whereas conference abstracts without full data and study protocols without results will not be included. Under Tier 2, to capture the full scope of evidence in this niche and emerging area of research, an inclusive approach will be applied. Both experimental and non-experimental studies are eligible, including RCTs, quasi-experiments (controlled before-after and non-randomized trials), prospective or retrospective cohort and cross-sectional studies, case-control studies, and qualitative studies (e.g. case studies, interviews, focus groups providing insight into experiences of PE in hospital schools), and mixed-methods studies ( Tenenbaum and Driscoll, 2005).

**Context**: The interventions of interest must take place in the context of a formal hospital school or in-hospital educational setting (e.g., any hospital-run educational services during inpatient care). As hospital schools exist in various forms around the world (e.g., well-established hospital school systems, ad hoc teaching by hospital staff or visiting teachers), no restriction will be based on country. Conversely context details (e.g., country, funding model of the hospital school, integration with the healthcare system) will be examined as these may influence implementation, and allow for cross-context variation analysis ( Pesce et al., 2021; Ciaccioni et al., 2025). Interventions delivered as part of the hospital-to-home transition or reintegration to regular school will be included only if they are explicitly linked with the hospital’s education program (for example, a transition PE plan that starts in hospital and continues after discharge as part of keeping the child engaged).

### 2.3 Data sources and search strategy

The following electronic bibliographic databases will be searched for reviews (Tier 1) and eligible studies (Tier 2):

• MEDLINE (via PubMed or Ovid)
• CINAHL (Cumulative Index to Nursing and Allied Health Literature)
• Embase (via Ovid)
• PsycINFO
• Cochrane Central Register of Controlled Trials (CENTRAL)
• Web of Science (Science Citation Index and Social Science Citation Index)
• Scopus
• EBSCOhost (which may include relevant education databases)

The search strategy will be tailored to each database, using combinations of keywords and subject headings related to: hospital schools (e.g. “hospital school”, “education in hospital”, “school reintegration”), physical education/activities (e.g. “physical education”, “exercise”, “physical activity”, “sports”, “play therapy”), children/adolescents, and hospital or inpatient context. Terms for systematic reviews will be also included when searching for Tier 1 (e.g. “systematic review” OR “meta-analysis” and filter by publication type). No language or publication date restrictions will be applied, with studies in any language and from all years considered for inclusion and translated where necessary. Other methods to identify relevant studies include: (i) reference checking and citation tracking, examining the reference lists of all included studies and relevant reviews for additional sources (backward citation searching) and forward citation tracking on key articles (e.g., via Web of Science or Google Scholar) to find more recent papers that cited those key studies; (ii) reports, policy documents, and guidelines from bodies such as ministries of education or health, international organisations (UNESCO, WHO), and networks like HOPE, searching websites and databases for education policies such as Wiki Youth ( https://national-policies.eacea.ec.europa.eu/youthwiki) and use targeted Google searches (e.g. “hospital school” AND “physical education” [country]”) to find relevant documents; (iii) expert consultation, leveraging the expertise of internal and external networks and collaborating researchers to ensure no key studies are missed. To ensure transparency, the full search strategy (see Table 1) for at least one major database (e.g., MEDLINE) will be published in an appendix of the final manuscript(s). Search results from all sources will be imported into reference management software, and duplicates will be removed prior to screening.

**Table 1.** The search terms and strategies.

Domain	Search terms
Setting and Context	(“Hospital Schools”[Mesh] OR “Child, Hospitalized”[Mesh] OR “Adolescent, Hospitalized”[Mesh] OR “hospital school*”[tw] OR “in-hospital education”[tw] OR “education in hospital”[tw] OR “hospital-based school*”[tw] OR “hospital classroom*”[tw] OR “hospital teaching”[tw] OR “hospital pedagogy”[tw] OR “school reintegration”[tw] OR “school re-entry”[tw] OR “school reentry”[tw] OR “school transition*”[tw] OR “hospital-to-school”[tw] OR “return to school”[tw] OR “return to learn*”[tw] OR “school liaison*”[tw])
Intervention	(“Physical Education and Training”[Mesh] OR “Exercise Therapy”[Mesh] OR “Play Therapy”[Mesh] OR “Motor Activity”[Mesh] OR “Exercise”[Mesh] OR “Physical Exertion”[Mesh] OR “Sports”[Mesh] OR “Physical Fitness”[Mesh] OR “Motor Skills”[Mesh] OR “Physical Activity”[tw] OR “physical education”[tw] OR “quality physical education”[tw] OR “exercise program*”[tw] OR “movement-based”[tw] OR “motor skill*”[tw] OR “adapted physical education”[tw] OR “sport*”[tw] OR “recreational activit*”[tw] OR “movement therap*”[tw] OR “therapeutic exercise”[tw] OR “active play”[tw] OR “structured play”[tw] OR “active break*”[tw] OR “active lesson*”[tw] OR “MVPA”[tw])
Population	(“Child”[Mesh] OR “Adolescent”[Mesh] OR “Pediatrics”[Mesh] OR “Students”[Mesh] OR child*[tw] OR pediatric*[tw] OR paediatric*[tw] OR adolescent*[tw] OR youth [tw] OR teen*[tw] OR student*[tw] OR pupil*[tw] OR “school-age”[tw] OR “school aged”[tw])

**Note.** The search includes both MeSH terms (e.g., “Physical Education and Training” **[Mesh]**) and free-text terms (e.g., “adapted physical education” **[tw]**) to increase sensitivity. Truncation symbols (e.g., *) ensure capture of variations (e.g., child, children; activity, activities). MeSH = Medical Subject Headings; MVPA = Moderate-to-Vigorous Physical Activity; tw = Text Word.

### 2.4 Use of artificial intelligence and automation tools

In line with current guidance on the responsible use of artificial intelligence in evidence synthesis ( Thomas et al., 2026), any automation or AI-enabled functionality will be used, if at all, only as supportive and non-decisional assistance within the review workflow. Core methodological decisions, including eligibility assessment, full-text inclusion or exclusion, data extraction, risk-of-bias or critical appraisal judgments, certainty assessment, and interpretation of findings, will remain under direct human responsibility and will be performed by the review team according to the procedures described below. If AI-enabled functions embedded within review platforms or reference-management software are used for tasks such as deduplication, record organization, or citation prioritization, all outputs will be fully verified by reviewers before any decision is made. The review team will document the name, version, developer, function, and timing of any such tool use, together with the safeguards adopted to ensure transparency, reproducibility, and human oversight.

### 2.5 Screening and study selection

All titles and abstracts resulting from the search will be screened for eligibility by at least two independent reviewers. To manage this process, a screening software or systematic review platform (e.g., Covidence or Rayyan) will be used. The reviewers will apply the inclusion criteria to each title/abstract; studies that clearly do not meet criteria will be excluded at this stage. For any title/abstract that appears potentially relevant, the full text will be retrieved. Full-text screening will likewise be done independently by two reviewers, who will read the articles in full and determine inclusion using a standardized form. Any disagreements or discrepancies between reviewers will be resolved through discussion and consensus, or by involving a third reviewer as an arbiter if necessary. Reasons for exclusion will be recorded at the full-text stage and a PRISMA flow diagram will be provided to depict the selection process. Where information is unclear or missing in a report (e.g., setting not certain), an attempt will be made to contact the study authors for clarification to make an inclusion decision.

### 2.6 Data extraction

A comprehensive data extraction form will be developed to capture all relevant information from the included studies and reviews. Prior to full data extraction, the form will be piloted on a few studies to ensure all reviewers have a consistent approach ( Khudair et al., 2022). Data extraction will be performed by two reviewers independently, with a consensus agreement procedure involving a third expert to reconcile any unsolvable disagreement. Key data items to extract from each included primary study (Tier 2) include: (i) publication details (e.g., authors, year, journal or source); (ii) study design (e.g., RCT, pre-post study, qualitative interview study); (iii) setting and context (e.g., country, type of hospital such as general or specialized children’s hospital, description of the hospital school, other relevant contextual factors); (iv) participant characteristics (e.g., number of participants, age range, diagnoses/health conditions, specific condition, length of stay); (v) intervention details (e.g., name or description of the PE or physical activity program, delivery expert such as teacher or therapist, frequency and duration, content and activities involved, equipment used, delivery modality such as individualized or group-based, intervention framed as part of curriculum or therapy); (vi) comparator details (e.g., standard care with no PE, or alternative activity); (vii) assessed outcomes, measurement instruments or methods used (e.g., fitness test, questionnaire, observation), and time-points of assessment; (viii) key results: outcomes data for each relevant measure (e.g., quantitative outcomes: numerical results such as mean and standard deviation, change scores, effect sizes, confidence intervals, p-values; qualitative outcomes: themes or illustrative quotes as reported); (ix) findings: summary of authors’ main conclusions regarding the effectiveness or impact of the intervention; (x) implementation/process info (e.g., notes on feasibility, adherence rates, drop-outs, barriers, facilitators observed, and any feedback from participants or staff ); (xi) funding and conflicts of interest of study authors (for risk of bias considerations).

For systematic reviews included in Tier 1, extracted data will include: (i) the review’s objective; (ii) the number of included studies; (iii) the population and intervention focus; (iv) key findings or meta-analytic result; and (v) the authors’ conclusions. Any overlapping studies between reviews will be mapped calculating the Corrected Covered Area (CCA) for all primary studies across the included systematic reviews ( Higgins et al., 2023).

For the policy documents, data extraction will involve capturing: (i) the source and type of document (e.g. law, guideline, policy memo) including the year of issue and if it is current or has been updated; (ii) the issuing body (ministry, organization); (iii) the geographic scope (e.g., regional, national, federal, international); (iv) the key content related to physical activity or PE in hospital schools (e.g. is PE mentioned as a requirement or recommendation? any standards for implementation? resources allocated?); and (v) any enforcement or implementation mechanisms described.

All extracted data will be cross-checked by the second reviewer to ensure accuracy. If any critical data are missing from a study, the authors will be contacted, when possible, to request additional information (e.g., if an outcome was measured but not fully reported).

### 2.7 Risk of bias and quality assessment

To assess the quality and internal validity of included studies, two reviewers will independently evaluate the risk of bias, with discrepancies resolved by consensus or third-party adjudication.

For RCTs, the Cochrane Risk of Bias 2.0 (RoB 2) tool will be used, evaluating bias across domains such as randomization process, deviations from intended interventions, missing outcome data, measurement of outcomes, and selection of reported results. For non-randomized intervention studies (controlled or uncontrolled), the ROBINS-I tool (Risk Of Bias In Non-randomized Studies of Interventions) will cover bias due to confounding, participant selection, classification of interventions, deviations from interventions, missing data, outcome measurement, and selection of reported results ( Sterne et al., 2016; Sterne et al., 2019).

For qualitative studies, the Critical Appraisal Skills Programme (CASP) checklist will be used to appraise qualitative study credibility (e.g., appropriate methodology, reflexivity, coherence of data) ( Long et al., 2020).

The methodological quality of the systematic reviews included in the umbrella review will be appraised using AMSTAR-2, assessing aspects like search comprehensiveness, clarity of inclusion criteria, risk of bias assessment, and appropriateness of conclusions ( Shea et al., 2017).

For the policy documents, a structured appraisal will be conducted using the AGREE-II tool. This framework will guide the assessment of methodological quality and reporting across key domains, including scope and purpose, stakeholder involvement, rigour of development, clarity of presentation, applicability, and editorial independence ( Brouwers et al., 2016).

Whilst each record’s appraisal will be summarized (e.g., “low risk of bias,” “some concerns,” or “high risk of bias” for RoB tools), these tools will not be used to outright exclude studies. Still, the risk of bias assessments will inform the interpretation of results and the GRADE assessment of evidence certainty.

### 2.8 Data synthesis and analysis

Initially, all studies will be summarized in a narrative format and in tables and figures, providing a descriptive overview of study characteristics (population, intervention types, settings) and results, by organizing findings according to key themes or outcome domains, grouping interventions by type (e.g., curriculum-based vs therapeutic exercise vs play activities) to compare their implementation and outcomes, and summarizing common barriers and facilitators reported across studies. The policy analysis results will be narrated separately and then integrated in the interpretation stage.

Where a sufficient number of homogeneous studies (in terms of intervention and outcome, and where statistical combination makes sense), meta-analyses will be conducted, using a random-effects model, as heterogeneity in study designs and populations is expected. Effect measures will be chosen based on outcome type: for dichotomous outcomes (e.g. proportion of children achieving a certain physical skill, or discharge rates), risk ratios or odds ratios with 95% confidence intervals will be used, whereas for continuous outcomes (e.g. fitness scores, quality of life ratings), mean differences will be calculated if studies use the same scale, or standardized mean differences if different scales are used. Moreover, if change-from-baseline scores are reported, those will be used in preference to post-intervention only, where possible ( Borenstein et al., 2021). Whilst missing data will be addressed by contacting study authors, if they remain still unavailable, available-case analysis will be applied discussing the potential impact of missing data on the findings of the present reviews ( Higgins et al., 2023). Heterogeneity across studies will be assessed quantitatively using the Chi-square test for heterogeneity (with a significance level of p < 0.10 indicating possible heterogeneity) and the I ^2^ statistic, with I ^2^ values of ~25%, ~50%, and ~ 75% considered low, moderate, and high heterogeneity respectively. The τ ^2^ (tau-squared) statistic from the random-effects models will be examined as an estimate of between-study variance ( Higgins et al., 2023; Ling et al., 2024). If substantial heterogeneity is detected, potential subgroup analyses will be explored to explain differences. A priori, potentially meaningful identified subgroups include: (i) participants’ age group (e.g., elementary/primary school children vs. middle/high school adolescents, since physical and cognitive development may influence outcomes); (ii) health condition category (e.g., children with oncology diagnoses vs. other chronic illnesses vs. acute conditions, as the nature of illness might affect ability to participate and benefit); (iii) intervention type (e.g., curriculum-based PE vs. therapeutic exercise vs. recreational play, to see if one modality shows different effects); (iv) professional delivering the intervention (e.g., primarily teacher-led vs. physiotherapist/health-professional-led vs. an interdisciplinary team, to examine whether the lead facilitator background impacts outcomes); (v) risk of bias of studies (e.g., higher-quality studies compared to lower-quality ones, to see if there’s a difference in estimated effects). Subgroup analyses will be performed by stratified meta-analyses (grouping studies by subgroup levels) and comparing the pooled estimates. Each study will contribute to only one subgroup in any given analysis, except possibly multi-arm studies which could contribute multiple comparisons. Sensitivity analyses will be also conducted to test the robustness of findings ( Higgins et al., 2023; Ling et al., 2024).

For qualitative data, a thematic synthesis will be performed, involving coding qualitative findings (e.g., interview quotes or authors’ thematic conclusions) and identifying common themes or concepts regarding experiences of PE in hospital schools (e.g., “PE as a relief from illness”, “Concerns about medical safety”, “Value of normality”). Importantly, the policy analysis findings will also be synthesized narratively and then integrated with the empirical evidence. This triangulation of policy and research data will highlight areas of convergence (e.g., policies supporting practices that evidence shows are beneficial), divergence (e.g., evidence recommends something not reflected in policy, or policy mandates something not supported by evidence), and gaps (areas neither well studied nor addressed in policy). By doing so, a coherent set of conclusions will be provided taking into account both what is being done (or should be done) in practice (policy perspective) and what works (evidence perspective).

### 2.9 Certainty of evidence assessment

The overall certainty of the evidence for key outcomes will be evaluated using the GRADE approach. For quantitative outcome domains (e.g. physical health outcomes), while the certainty will be by default higher for RCT evidence and lower for observational, factors that can downgrade (risk of bias, inconsistency, indirectness, imprecision, and publication bias) or occasionally upgrade (for large effect, dose-response, etc.) will be evaluated. For bodies of evidence that include mixed study types, the overall contribution of each will be considered. For qualitative evidence, the GRADE-CERQual (Confidence in the Evidence from Reviews of Qualitative research) approach will be applied to assess confidence in synthesized qualitative findings, examining methodological limitations, coherence, adequacy of data, and relevance ( Lewin et al., 2018; Brozek et al., 2021; Kolovelonis et al., 2024). Summary tables will be presented indicating the confidence (high, moderate, low, very low) in the evidence for major outcome categories, along with rationales for any downgrading. Where quantitative and qualitative findings are combined, the confidence will be described narratively.

## 3 Discussion

Evidence indicates variability and, in some cases, limited provision of PE and physical activity opportunities for children in hospital schools, as reported by educators, healthcare providers, and policymakers ( Issaka and Hopkins, 2015; West et al., 2019; Cistulli et al., 2025). At the policy level, international initiatives such as the ‘Hospital Schools’ Guidelines on Physical Education’ (HOPE) project ( European Commission, 2026) frame PE as an important component of educational provision in hospital settings and advocate for its accessibility, inclusivity, and positive delivery across different health and educational contexts. In fact, hospitalised young people deserve access to the same holistic education – including physical development – that their healthy peers receive, yet practical and systemic barriers have led to inconsistencies in delivering PE in these settings ( Stodden et al., 2023; Catenazzo, 2024; Stodden and Pesce, 2025). This protocol outlines the first comprehensive effort to systematically review the landscape of PE in hospital schools. By collating evidence from individual intervention studies and higher-level reviews, and by examining the governing policy frameworks, this review aims to address a critical gap in the literature and practice.

One key contribution of this study will be an improved understanding of what works in engaging hospitalised students in physical activity ( Braam et al., 2016; Crowder et al., 2022). The review will identify strategies that have been successful in different contexts such as types of activities that are well-tolerated by patients, or teaching approaches that motivate students despite medical challenges, as well as insights to educate PE teachers ( Standage et al., 2005; Fernandez-Rio and Iglesias, 2024). It will also shed light on feasibility aspects: what are the common obstacles (e.g., infection control concerns, lack of space) and what solutions have been tried to overcome them? By gathering reports of barriers and facilitators across studies, the review can inform practical guidelines for hospital school staff on how to implement or adapt PE programs (e.g., scheduling considerations, necessary training for staff, adaptations for various medical conditions) ( World Health Organization, 2010; Shields et al., 2012). The inclusion of qualitative evidence is especially valuable here, as it can capture the experiences and perceptions of students and teachers, information that pure outcome measures might miss ( Green and Thorogood, 2018; Martins et al., 2021). By systematically examining available studies, the review will clarify whether PE interventions are associated with improvements in physical functioning, psychological well-being, or academic engagement, thereby providing empirical support for investment in such programs. Equally important, the review will identify outcomes showing inconsistent effects or potential risks, informing cautious and context-sensitive implementation ( McLennan and Thompson, 2015).

By conducting an umbrella review, the study situates hospital school PE within the broader landscape of paediatric hospital-based physical activity interventions ( Rustler et al., 2017; Baldwin et al., 2020). This approach enables comparison between outcomes associated with hospital school–led PE and those reported for other in-hospital activity programs ( Runco et al., 2019; Scheffers et al., 2021). It also facilitates the identification of evidence gaps, for example when strong evidence exists for benefits in specific patient groups at the hospital level, but corresponding PE interventions within hospital school settings are absent ( Theobald et al., 2025). The policy framework analysis extends the relevance of this work beyond academia by mapping existing regulations and guidance ( McLennan and Thompson, 2015). This analysis is expected to reveal gaps or inconsistencies in current policies, which may help explain the variability of practice across jurisdictions, while also identifying examples of more structured approaches that could inform policy development elsewhere ( Amornsriwatanakul et al., 2025). Overall, the findings will support evidence-informed recommendations for educational authorities and hospital administrators aimed at strengthening and standardising PE provision for hospitalised students ( Kohl III et al., 2013; Longmuir et al., 2013; American Public Health Association, 2021; World Health Organization, 2022).

Several challenges and limitations need to be acknowledged. First, the evidence base may be limited in quantity and quality. Hospital school research is a recently developing field; relatively few rigorous CTs are anticipated, and many included studies might be descriptive with small sample sizes. This could limit the possibility to perform meta-analysis or draw strong conclusions ( Borenstein et al., 2021). Following GRADE methodology, it is possible that the overall certainty of evidence for some outcomes will be low ( Brozek et al., 2021). This in itself is a finding, highlighting the need for more robust research in the future. Second, there will likely be substantial heterogeneity across studies – in patient populations (ranging from oncology to psychiatry), intervention types (from structured PE classes to casual play), and outcome measures. Such heterogeneity can complicate synthesis; mitigation measures will include stratifying analyses and using narrative approaches where meta-analysis is not appropriate ( Higgins et al., 2023; Lee et al., 2025). Third, as non-randomized and qualitative studies are eligible for inclusion, there is a risk of bias in the evidence (e.g. positive publication bias, or studies with null results not published), which should be reduced by means of comprehensive searches including all available languages, and will be explicitly discussed when interpreting results ( Sterne et al., 2016; Long et al., 2020). A further limitation concerns the policy analysis. Policy documents may be difficult to compare across legal contexts and languages, may be inaccessible, or may exist only as internal or unpublished guidance ( Browne et al., 2019). To mitigate this, expert networks and grey literature searches will be used to identify influential documents. Potential subjectivity in interpretation will be addressed through independent review by multiple reviewers and a focus on clearly defined, objective policy features. Any recommendations will need to be context-sensitive, as approaches effective in well-resourced settings may not be directly transferable to lower-resourced contexts. Accordingly, the discussion will emphasise underlying principles and adaptable strategies rather than uniform solutions ( McLennan and Thompson, 2015). Despite these challenges, the approach is strengthened by its broad scope and interdisciplinary integration ( European Commission, 2026). By combining evidence synthesis with policy analysis and drawing on expertise in education, health, and systematic review methodology, the study aims to produce findings that are both methodologically robust and relevant to practice. Adherence to established reporting standards, including PRISMA-P and PROSPERO, will further support transparency and reproducibility ( Shamseer et al., 2015).

In conclusion, this study will provide a comprehensive overview of PE in hospital schools, clarifying its role and informing effective implementation. The findings are expected to support improvements in practice, staff training, resource allocation, and policy development, thereby contributing to more inclusive and high-quality educational provision for hospitalised children.

## Ethics and dissemination

This multi-tiered review (encompassing a systematic review, umbrella review, and policy framework analysis) will rely exclusively on secondary data from published studies and publicly available policy documents. No primary data collection involving human participants will be conducted, and thus no formal ethics approval is required.

The findings of this review will be disseminated through multiple channels. We intend to publish the results in peer-reviewed journals and present them at relevant national and international conferences. Furthermore, key insights will be communicated directly to policy and practice stakeholders – for example, hospital school educators, healthcare providers, and education/health policymakers – via targeted presentations or briefings. Through these dissemination avenues, we aim to ensure that the evidence generated is accessible to the academic community and informs policy development and practical guidelines for implementing physical education in hospital school settings.

## Data Availability

The manuscript contains the following Extended Data: Ciaccioni, S., Pesce, C., Benzing, V., Schmidt, M., Magalhães, P., Rosário, P., Pedrosa Leal, L., Pontes, C. M., Oddi, L., Betello, F., Bradic, S., & Capranica, L. (2026). PRISMA-P for the Manuscript: MSCA HOPE - Physical Education in Hospital Schools: A Protocol Paper for Systematic and Umbrella Reviews with Policy Framework Analysis. Zenodo. https://doi.org/10.5281/zenodo.20187448 Data are available under the terms of the Creative Commons Zero “No rights reserved” data waiver ( CC0 1.0 Public domain dedication).

## References

[ref1] American Public Health Association: *Supporting Physical Education in Schools for All Youth.* Washington, DC, USA: American Public Health Association;2021.

[ref2] Amornsriwatanakul A Chinapong S Wattanapisit A : Thailand National Physical Activity Promotional and Action Plans for Children and Youth: Development and Implementation Challenges. *J Phys Act Health.* 2025;22:1453–1463. 10.1123/jpah.2025-0083 40846306

[ref3] Baldwin CE Parry SM Norton L : A scoping review of interventions using accelerometers to measure physical activity or sedentary behaviour during hospitalization. *Clin Rehabil.* 2020;34:1157–1172. 10.1177/0269215520932965 32517508

[ref4] Benzing V Spitzhuttl J Siegwart V : Effects of Cognitive Training and Exergaming in Pediatric Cancer Survivors-A Randomized Clinical Trial. *Med Sci Sports Exerc.* 2020;52:2293–2302. 10.1249/MSS.0000000000002386 33064404 PMC7556245

[ref5] Biddle SJ Ciaccioni S Thomas G : Physical activity and mental health in children and adolescents: An updated review of reviews and an analysis of causality. *Psychol Sport Exerc.* 2019;42:146–155. 10.1016/j.psychsport.2018.08.011

[ref6] Borenstein M Hedges LV Higgins JP : *Introduction to meta-analysis.* John Wiley & Sons;2021.

[ref7] Bowen GA : Document analysis as a qualitative research method. *Qualitative research journal.* 2009;9:27–40. 10.3316/QRJ0902027

[ref8] Braam KI Van Der Torre P Takken T : Physical exercise training interventions for children and young adults during and after treatment for childhood cancer. *Cochrane Database Syst Rev.* 2016;3:CD008796. 27030386 10.1002/14651858.CD008796.pub3 PMC6464400

[ref9] Brouwers MC Kerkvliet K Spithoff K : The AGREE Reporting Checklist: a tool to improve reporting of clinical practice guidelines. *BMJ.* 2016;352:i1152. 26957104 10.1136/bmj.i1152 PMC5118873

[ref10] Brown P Brunnhuber K Chalkidou K : How to formulate research recommendations. *BMJ.* 2006;333:804–806. 10.1136/bmj.38987.492014.94 17038740 PMC1602035

[ref11] Browne J Coffey B Cook K : A guide to policy analysis as a research method. *Health Promotion International.* 2019;34:1032–1044. 10.1093/heapro/day052 30101276

[ref12] Brozek JL Canelo-Aybar C Akl EA : GRADE Guidelines 30: the GRADE approach to assessing the certainty of modeled evidence-An overview in the context of health decision-making. *J Clin Epidemiol.* 2021;129:138–150. 32980429 10.1016/j.jclinepi.2020.09.018 PMC8514123

[ref13] Bull FC Al-Ansari SS Biddle S : World Health Organization 2020 guidelines on physical activity and sedentary behaviour. *Br J Sports Med.* 2020;54:1451–1462. 10.1136/bjsports-2020-102955 33239350 PMC7719906

[ref14] Catenazzo T : *La scuola in ospedale e l'istruzione domiciliare: formazione degli insegnanti e linee di indirizzo nazionali [in Italian].* Carocci;2019.

[ref15] Catenazzo T : *Quando la scuola è di casa: Prassi inclusive ed educazione di prossimità nel lavoro del docente ospedaliero e domiciliare [in Italian].* Mimesis;2024.

[ref16] Ciaccioni S Compernolle S Lerfald M : Modifiable determinants of older adults' physical activity and sedentary behavior in community and healthcare settings: a DE-PASS systematic review and meta-analysis. *Eur Rev Aging Phys Act.* 2025;22:9. 10.1186/s11556-025-00373-y 40413376 PMC12103017

[ref17] Ciaccioni S Pesce C Benzing V : PRISMA-P for the Manuscript: MSCA HOPE - Physical Education in Hospital Schools: A Protocol Paper for Systematic and Umbrella Reviews with Policy Framework Analysis. *Zenodo.* 2026. 10.5281/zenodo.20187448

[ref18] Cistulli M Bellucci M Casella R : Educazione fisica nella scuola in ospedale *Percorsi didattici ventennali [In Italian].* Calzetti & Mariucci Ed;2025.

[ref19] Commission, E., Education, E., and Agency, C.E: *Physical education and sport at school in Europe.* Publications Office of the European Union;2013.

[ref20] Commission, E., Education, E., and Agency, C.E: *The situation of young people in the European Union – EU youth report 2024.* Publications Office of the European Union;2025.

[ref21] Crowder SL Buro AW Stern M : Physical activity interventions in pediatric, adolescent, and young adult cancer survivors: a systematic review. *Support Care Cancer.* 2022;30:4635–4649. 10.1007/s00520-022-06854-5 35064822 PMC9175508

[ref22] Davies D Hartfield D Wren T : Children who 'grow up' in hospital: Inpatient stays of six months or longer. *Paediatr Child Health.* 2014;19:533–536. 10.1093/pch/19.10.533 25587232 PMC4276387

[ref23] Dixon CE : *Teaching Physical Education in Nontraditional Settings [Doctoral Thesis].* Auburn University;2020.

[ref24] European Commission: *2024 Annual Work Programme “Erasmus+”: the Union Programme for Education, Training, Youth and Sport.* European Commission;2023.

[ref25] European Commission: Hospital schools' guidelines On Physical Education (HOPE): An International, Interdisciplinary and Intersectoral Endeavour. 2026. [Accessed]. Reference Source

[ref26] European Union: Guidelines recommended policy actions in support of health-enhancing physical activity. EU Working Group “Sport & Health”. 2008.

[ref27] Fernandez-Rio J Iglesias D : What do we know about pedagogical models in physical education so far? An umbrella review. *Physical Education and Sport Pedagogy.* 2024;29:190–205. 10.1080/17408989.2022.2039615

[ref28] Gold JM Hall M Shah SS : Long length of hospital stay in children with medical complexity. *J Hosp Med.* 2016;11:750–756. 10.1002/jhm.2633 27378587

[ref29] Green J Thorogood N : *Qualitative methods for health research.* 2018. 10.4135/9781036234386

[ref30] Higgins J Thomas J Chandler J : *Cochrane handbook for systematic reviews of interventions version 6.4.* Cochrane;2023. [Accessed]. Reference Source

[ref31] Issaka A Hopkins L : Introducing physical education to hospital learning–can patients participate?. *Asia-Pacific Journal of Health, Sport and Physical Education.* 2015;6:23–40. 10.1080/18377122.2014.997859

[ref32] Jiliberto F Zarate Alva N : Influencing Factors In-Hospital School Education: Exploring the Context From the Teacher's Perspective. *Contin Educ.* 2024;6:1–21. 39897589 10.5334/cie.126 PMC11784520

[ref33] Khudair M Marcuzzi A Ng K : DE-PASS Best Evidence Statement (BESt): modifiable determinants of physical activity and sedentary behaviour in children and adolescents aged 5-19 years-a protocol for systematic review and meta-analysis. *BMJ Open.* 2022;12:e059202. 10.1136/bmjopen-2021-059202 PMC9490573 36127107

[ref34] Kohl HW III Cook HD Activity, C.O.P : The Effectiveness of Physical Activity and Physical Education Policies and Programs: Summary of the Evidence. *Educating the Student Body: Taking Physical Activity and Physical Education to School.* National Academies Press (US);2013. 24851299

[ref35] Kolovelonis A Syrmpas I Marcuzzi A : DE-PASS best evidence statement (BESt): determinants of adolescents' device-based physical activity and sedentary behaviour in settings: a systematic review and meta-analysis. *BMC Public Health.* 2024;24:1706. 10.1186/s12889-024-19136-y 38926707 PMC11202347

[ref36] Lee Y Guidotti F Capranica L : Olympic combat sports and mental health in children and adolescents with disability: A systematic review of controlled trials. *Front Psychol.* 2025;16:1567978. 10.3389/fpsyg.2025.1567978 40376492 PMC12078338

[ref37] Lewin S Booth A Glenton C : Applying GRADE-CERQual to qualitative evidence synthesis findings: introduction to the series. *Implement Sci.* 2018;13:2. 10.1186/s13012-017-0688-3 29384079 PMC5791040

[ref38] Ling FCM Khudair M Ng K : DE-PASS Best Evidence Statement (BESt): Determinants of self-report physical activity and sedentary behaviours in children in settings: A systematic review and meta-analyses. *PLoS One.* 2024;19:e0309890. 10.1371/journal.pone.0309890 39585854 PMC11588252

[ref39] Long HA French DP Brooks JM : Optimising the value of the critical appraisal skills programme (CASP) tool for quality appraisal in qualitative evidence synthesis. *Research Methods in Medicine & Health Sciences.* 2020;1:31–42. 10.1177/2632084320947559

[ref40] Longmuir PE Brothers JA De Ferranti SD : Promotion of physical activity for children and adults with congenital heart disease: a scientific statement from the American Heart Association. *Circulation.* 2013;127:2147–2159. 23630128 10.1161/CIR.0b013e318293688f

[ref41] Ludgério MJB Pontes CM Dos Santos BLC : Pedagogical practices developed with children through hospital classes: An integrative literature review. *Journal of Pediatric Nursing-Nursing Care of Children & Families.* 2023;72:E10–E18. 10.1016/j.pedn.2023.05.014 37302968

[ref42] Martins J Costa J Sarmento H : Adolescents' Perspectives on the Barriers and Facilitators of Physical Activity: An Updated Systematic Review of Qualitative Studies. *Int J Environ Res Public Health.* 2021;18:4954. 10.3390/ijerph18094954 34066596 PMC8125166

[ref43] Marusak HA Iadipaolo AS Cohen C : Martial Arts-Based Therapy Reduces Pain and Distress Among Children with Chronic Health Conditions and Their Siblings. *Journal of Pain Research.* 2020;Volume 13:3467–3478. 10.2147/JPR.S283364 PMC7778380 33402843

[ref44] Mclennan N Thompson J : *Quality physical education (QPE): Guidelines for policy makers.* Unesco Publishing;2015.

[ref45] Metzler MW Mckenzie TL Van Der Mars H : Health optimizing physical education (HOPE): A new curriculum for school programs—Part 2: Teacher knowledge and collaboration. *Journal of Physical Education, Recreation & Dance.* 2013;84:25–34. 10.1080/07303084.2013.779531

[ref46] Pagnani B Caldoro S Guidi A : *Scuola in ospedale. Una formazione di qualità per integrare benessere-apprendimento-salute [In Italian].* Firenze: Le Monnier;2004.

[ref47] Parliament, E Union, D.-G.F.I.P.O.T : *The fight against cancer is a team sport – The role of education and sport – Workshop Brussels, 13 July 2016 – Proceedings.* European Parliament;2016.

[ref48] Pesce C Vazou S Benzing V : Effects of chronic physical activity on cognition across the lifespan: a systematic meta-review of randomized controlled trials and realist synthesis of contextualized mechanisms. *Int Rev Sport Exerc Psychol.* 2021;16(1):722–760. 10.1080/1750984x.2021.1929404

[ref49] Reid TR : *The healing of America: A global quest for better, cheaper, and fairer health care.* Penguin;2010.

[ref50] Runco DV Yoon L Grooss SA : Nutrition & Exercise Interventions in Pediatric Patients with Brain Tumors: A Narrative Review. *J Natl Cancer Inst Monogr.* 2019;2019:163–168. 10.1093/jncimonographs/lgz025 31532532

[ref51] Rustler V Hagerty M Daeggelmann J : Exercise interventions for patients with pediatric cancer during inpatient acute care: A systematic review of literature. *Pediatr Blood Cancer.* 2017;64:e26567. 10.1002/pbc.26567 28423225

[ref52] Santos SDS Moussalle LD Heinzmann-Filho JP : Effects of Physical Exercise during Hospitalization in Children and Adolescents with Cancer: A Systematic Review. *Rev Paul Pediatr.* 2020;39:e2019313. 33027320 10.1590/1984-0462/2021/39/2019313 PMC7537404

[ref53] Scheffers LE Berg L Ismailova G : Physical exercise training in patients with a Fontan circulation: A systematic review. *Eur J Prev Cardiol.* 2021;28:1269–1278. 10.1177/2047487320942869 34551076

[ref54] Shamseer L Moher D Clarke M : Preferred reporting items for systematic review and meta-analysis protocols (PRISMA-P) 2015: elaboration and explanation. *BMJ.* 2015;350:g7647. 10.1136/bmj.g7647 25555855

[ref55] Shea BJ Reeves BC Wells G : AMSTAR 2: a critical appraisal tool for systematic reviews that include randomised or non-randomised studies of healthcare interventions, or both. *BMJ.* 2017;358. 10.1136/bmj.j4008 PMC5833365 28935701

[ref56] Shields N Synnot AJ Barr M : Perceived barriers and facilitators to physical activity for children with disability: a systematic review. *British journal of sports medicine.* 2012;46:989–997. 10.1136/bjsports-2011-090236 21948121

[ref57] Standage M Duda JL Ntoumanis N : A test of self-determination theory in school physical education. *British journal of educational psychology.* 2005;75:411–433. 10.1348/000709904X22359 16238874

[ref58] Sterne JA Hernan MA Reeves BC : ROBINS-I: a tool for assessing risk of bias in non-randomised studies of interventions. *BMJ.* 2016;355:i4919. 10.1136/bmj.i4919 27733354 PMC5062054

[ref59] Sterne JC Savovic J Page MJ : RoB 2: a revised tool for assessing risk of bias in randomised trials. *BMJ.* 2019;366:l4898. 31462531 10.1136/bmj.l4898

[ref60] Stodden DF Pesce C : Thinking Outside the Box and Exploring the Infinity Within the Box: Suggestions for Advancing Holistic Development Research With Movement at Its Core. *J Mot Learn Dev.* 2025;1:1–9.

[ref61] Stodden DF Pesce C Zarrett N : Holistic Functioning from a Developmental Perspective: A New Synthesis with a Focus on a Multi-tiered System Support Structure. *Clin Child Fam Psychol Rev.* 2023;26:343–361. 10.1007/s10567-023-00428-5 36826703

[ref62] Tenenbaum G Driscoll MP : *Methods of research in sport sciences: Quantitative and qualitative approaches.* Meyer & Meyer Verlag;2005.

[ref63] Theobald U Mayer J Giese M : Physical education for students with chronic health conditions: A scoping review. Robert Langnickel, Annett Thiele, Nicola. 2025;228.

[ref64] Thomas J Hair K Noel-Storr A : Responsible use of AI in Evidence Synthesis (RAISE 2026): building and evaluating AI evidence synthesis tools (version 3; updated 13 March 2026). 2026.

[ref65] Unesco: International charter of physical education, physical activity and sport. 2015.

[ref66] West SL Banks L Schneiderman JE : Physical activity for children with chronic disease; a narrative review and practical applications. *BMC Pediatr.* 2019;19:12. 10.1186/s12887-018-1377-3 30621667 PMC6325687

[ref67] World Health Organization: Global recommendations on physical activity for health. 2010. 26180873

[ref68] World Health Organization: WHO guidelines on physical activity and sedentary behaviour: web annex: evidence profiles. 2020. 33369898

[ref69] World Health Organization: Global status report on physical activity 2022: web annex: global action plan on physical activity monitoring framework, indicators and data dictionary. *Global status report on physical activity 2022: web annex: global action plan on physical activity monitoring framework, indicators and data dictionary.* 2022.

