# MSCA HOPE - Physical education in hospital schools: A protocol paper for systematic and umbrella reviews with policy framework analysis

**DOI:** 10.12688/openreseurope.23993.2

**Published:** 2026-08-27

**Authors:** Simone Ciaccioni, Caterina Pesce, Valentin Benzing, Mirko Schmidt, Paula Magalhães, Pedro Rosário, Luciana Pedrosa Leal, Cleide Maria Pontes, Livia Oddi, Filippo Betello, Slaviša Bradić, Laura Capranica

**Affiliations:** 1 Department of Movement, Human and Health Sciences, University of Rome “Foro Italico”, Rome, Italy, 00135, Italy; 2 Department of Kinesiology, Michigan State University, East Lansing, MI, 48824, USA; 3 Institute of Sport Science, Department of Sport Pedagogy, University of Bern, Bern, 3012, Switzerland; 4 School of Psychology, University of Minho, Braga, 4710-057, Portugal; 5 Department of Nursing, Federal University of Pernambuco, Recife, PE, 50670-420, Brazil; 6 Department of Information Engineering, Control and Management, Sapienza University of Rome, Rome, 00185, Italy; 7 Department of Computer, Control and Management Engineering, Sapienza University of Rome, Rome, 00185, Italy; 8 International Judo Federation Academy Foundation, Malta, XBX 1421, Malta

**Keywords:** sport science, hospitalised youth, systematic literature review, barriers and facilitators, interprofessional collaboration, physical activity programs, health–education integration, evidence-informed policy.

## Abstract

Hospital school programs support educational continuity for children and adolescents during hospitalization, but the provision of physical education (PE) remains inconsistent and is rarely addressed by unified standards. This protocol aims to describe the methods for three complementary components designed to synthesise evidence on PE and related movement interventions in hospital settings and to map the policy frameworks governing PE provision in hospital schools. Tier 1 is an umbrella review of existing systematic reviews on physical activity interventions for hospitalised youth. Tier 2 is a systematic review of primary studies examining PE in hospital-school settings. For Tier 2, PE is operationally defined as a planned movement-based learning experience with an explicit educational purpose that is formally integrated into the hospital-school program or involves educational staff in its planning, delivery or assessment. Therapeutic exercise, rehabilitation and active play without this educational connection will be excluded from Tier 2, although they may be represented in Tier 1. A parallel policy framework analysis will identify and compare relevant laws, regulations, curricula, strategies and guidelines. Separate search strategies will be used for each component across health, education, sport and policy sources, without language or date restrictions. Narrative synthesis will be the primary approach. Meta-analysis will be considered only when at least three independent studies are sufficiently comparable in population, intervention, design, outcome construct and assessment time point. Priority synthesis will distinguish implementation/process evidence from effectiveness outcomes. Safety, implementation and educational participation will be synthesised primarily using narrative, qualitative and mixed-method approaches, whereas physical functioning and psychosocial well-being will be prioritised for quantitative effect synthesis and Grading of Recommendations Assessment, Development and Evaluation (GRADE) when sufficiently comparable evidence is available. The planned reviews are intended to clarify the available evidence and policy landscape and may inform subsequent guideline development for PE in hospital schools. PROSPERO registration: CRD420251156215.

## Plain Language summary

Children and adolescents who spend time in hospital often continue their education through hospital school programmes. These programmes help them stay connected with learning, teachers, and classmates. However, physical education is not always included.

This is important because movement, play, exercise, and physical education can support children’s physical health, emotional wellbeing, social participation, and learning. In hospital settings, however, physical education can be difficult to organise. Children may have different health conditions, energy levels, treatment schedules, and safety needs. Hospitals and schools may also follow different rules, and there are no shared standards to guide practice.

This study will bring together the best available knowledge on physical education and physical activity for children and adolescents in hospital settings. The researchers will review previous research studies and examine existing policies and guidelines from different countries and organisations. They will look at what has been studied, what seems helpful, what evidence is still missing, and what rules or recommendations already exist.

The study will consider outcomes related to physical health, mental and social wellbeing, learning, clinical conditions, and practical delivery. It will also assess how reliable the available evidence is.

The results will help build a clearer evidence base for future guidance on physical education in hospital schools. The long-term aim is to support teachers, healthcare professionals, schools, families, and policymakers in offering safe, meaningful, and inclusive physical education opportunities for children and adolescents during hospitalisation.

This review protocol is registered in PROSPERO: CRD420251156215.

## 1. Introduction

Hospital schools provide educational services to children and adolescents whose treatment interrupts regular schooling, helping to preserve learning, peer connection and a sense of normality during hospitalization ( Issaka and Hopkins, 2015; Jiliberto and Zarate Alva, 2024). Whilst form and resourcing of these services vary across health and education systems, physical education (PE) is frequently omitted because of limited space, equipment, specialist staff and time, together with concerns about medical safety and coordination with clinical care ( Catenazzo, 2019; Catenazzo, 2024; Cistulli et al., 2025).

PE is a curriculum area with explicit learning aims related to movement competence, knowledge and understanding, meaningful participation, and personal and social development ( McLennan and Thompson, 2015; Opstoel et al., 2020; GloPL Consortium, 2026). It is therefore conceptually distinct from physical activity as a broad behaviour of bodily movement resulting in energy expenditure ( World Health Organization, 2022; Ciaccioni et al., 2026a), from therapeutic exercise or rehabilitation prescribed primarily to address symptoms, impairments, functioning, or other health-related needs ( Taylor et al., 2007; Gutenbrunner et al., 2011) and from recreational or play-based movement that may occur without explicit curricular learning objectives ( Pellegrini and Smith, 1998; Martins et al., 2021). Although these activities may overlap in hospital settings, they differ in their primary purposes, intended learning or clinical outcomes, and the professional responsibilities associated with their planning and delivery. Consequently, evidence from clinical exercise programs can inform, but cannot automatically be interpreted as evidence for PE provision within hospital schools.

Research on hospital-based movement is dispersed across education, paediatric rehabilitation, psychology, nursing and sport sciences. Existing reviews have generally examined physical activity or therapeutic exercise in specific clinical groups rather than PE as an educational provision ( Dixon, 2020; Ludgério et al., 2023; Theobald et al., 2025). In parallel, international commitments support inclusive education and participation for hospitalised children, but the extent to which laws, curriculum frameworks and implementation guidance address PE in hospital schools has not been systematically mapped ( McLennan and Thompson, 2015; UNESCO, 2015; European Parliament, 2016; European Commission, 2025). Consequently, the evidence base and the policy environment remain difficult to interpret together.

In response to these gaps, a multi-component review has been designed to consolidate evidence and policy information on PE in hospital schools. The objectives are to: (i) identify and describe the characteristics, and delivery models of PE interventions in hospital-school settings; (ii) summarise evidence concerning their safety, feasibility and physical, psychosocial, and educational outcomes; (iii) synthesize systematic-review evidence on broader hospital-based physical activity interventions that may offer transferable insights; (iv) map international, national and regional policy and regulatory provisions relevant to PE in hospital schools, including professional preparation and intersectoral responsibilities; (v) integrate empirical and policy findings to identify areas of convergence, divergence and uncertainty.

Thus, developed within the framework of the MSCA Global Postdoctoral Fellowship project “Hospital schools’ guidelines On Physical Education” (HOPE; https://cordis.europa.eu/project/id/101206417), this protocol is intended to provide an evidence-informed foundation for later guideline development.

## 2 Methods and analysis

### 2.1 Review structure

The present protocol was prospectively registered in the International Prospective Register of Systematic Reviews (PROSPERO) on 04 December 2025 (registration number: CRD420251156215). The preparation and reporting of this protocol manuscript were conducted in accordance with the Preferred Reporting Items for Systematic Review and Meta-Analysis Protocols (PRISMA-P; see Extended Data: ( Ciaccioni et al., 2026b)) guidelines ( Shamseer et al., 2015). The plan comprises two evidence-synthesis tiers and a parallel policy framework analysis. The components will be conducted separately using purpose-specific eligibility criteria, searches, appraisal methods and syntheses, and will be integrated only at the interpretation stage ( Tenenbaum and Driscoll, 2005; Bowen, 2009; Browne et al., 2019).

• Tier 1: Umbrella review of systematic reviews. This component will include systematic reviews with or without meta-analyses, that use explicit eligibility criteria and reproducible search methods to examine physical activity, exercise, and movement-based interventions delivered to hospitalised children and adolescents. Its purpose is to summarise higher-level evidence on benefits, harms, and implementation considerations that may be relevant, but not necessarily directly transferable, to hospital-school PE.
• Tier 2: Systematic review of primary studies. This component will include quantitative, qualitative, and mixed-methods primary studies of PE in formal hospital school or hospital-associated educational programs. Because the field is expected to be small, eligible designs will include randomised and non-randomised studies, observational studies, qualitative studies, mixed-methods studies, and case reports or series that provide sufficient information on a structured PE intervention or its implementation. Descriptive and case-based evidence will be used for mapping and narrative interpretation rather than pooling estimates of effectiveness.
• Policy framework analysis. A structured document analysis will identify binding and non-binding instruments relevant to PE or movement provision in hospital education. Eligible sources will include legislation, regulations, ministerial decrees or circulars, curriculum frameworks, national or regional strategies, official standards, implementation guidance and international normative or recommendation documents. Expert consultation will be used only to identify potentially missing documents; expert opinions will not be analysed as data.

**Figure 1.**
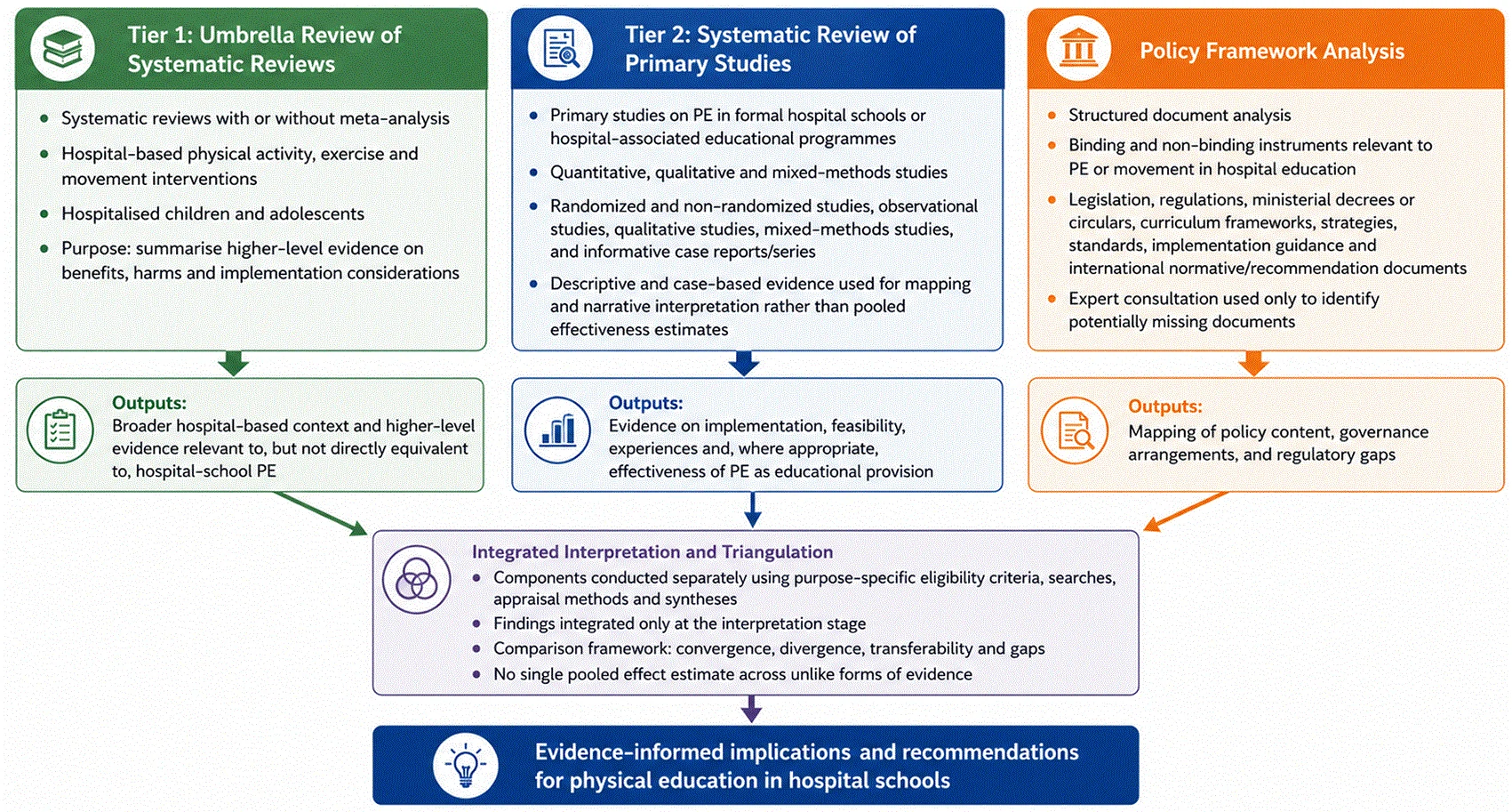
Overview of the concurrent multi-tier design and evidence integration. Note: PE: Physical education; PROSPERO: International Prospective Register of Systematic Reviews; PRISMA-P: Preferred Reporting Items for Systematic Review and Meta-Analysis Protocols.

Figure 1 presents the concurrent design. Tier 1 provides the broader hospital-based physical activity context, Tier 2 focuses specifically on PE as educational provision in hospital-school settings, and the policy analysis examines the governance context. Findings will be compared through a predefined integration framework addressing convergence, divergence, transferability and gaps, without combining unlike forms of evidence into a single effect estimate.

### 2.2 Eligibility criteria

Eligibility criteria were developed using the Population, Intervention, Comparator, Outcomes and Study design (PICOS) framework, with an additional Context criterion ( Brown et al., 2006). The terminology and Tier 2 screening decision rule are presented in Table 1. The definitions are intended to prevent educational PE from being conflated with clinical treatment or unstructured recreation while allowing transparent assessment of interventions that combine educational and therapeutic elements.

**Table 1.** Operational terminology and Tier 2 screening decision rule.

Term	Operational meaning in this protocol	Tier 2 eligibility
Physical education	Planned movement-based learning with explicit educational aims related to curriculum, movement competence, knowledge, participation, self-regulation, or personal and social development.	Eligible when the educational purpose is explicit and the activity is either formally integrated into the hospital-school program or involves educational staff in planning, delivery or assessment.
Physical activity	Any bodily movement that increases energy expenditure, irrespective of purpose or setting.	Eligible only when it also meets the PE educational decision rule. Otherwise, it may be relevant to Tier 1.
Exercise or therapeutic exercise	Planned and structured activity undertaken primarily to improve fitness, symptoms, function or health.	Eligible only when it has an explicit educational purpose and a documented hospital-school connection. Clinically prescribed exercise alone is excluded.
Rehabilitation or physiotherapy	Clinical assessment and treatment intended to restore or maintain function.	Excluded when delivered solely as healthcare. Included only when formally integrated with educational aims and the hospital-school program.
Active play or recreation	Enjoyable spontaneous or structured movement primarily intended for play, leisure or social interaction.	Eligible only when educational objectives and hospital-school integration are documented.
Borderline cases	Activities combining educational, therapeutic and recreational purposes.	Criterion A, explicit educational purpose, is mandatory. In addition, criterion B, formal hospital-school integration, or criterion C, documented educational-staff involvement, must be present. Unclear cases will undergo duplicate full-text assessment and author contact where feasible.

*Note*. PE: Physical education.

**Population and context**: Tier 2 will include children and adolescents of compulsory or upper-secondary school age, approximately 5 to 18 years, who receive education during inpatient care or through a hospital-associated educational program. All diagnoses, treatment pathways and countries will be eligible. Home or outpatient education will be excluded unless it forms part of a documented hospital-school transition or reintegration program. Tier 1 will include systematic reviews of hospitalised children and adolescents, whether or not the intervention is connected to schooling.

**Interventions**: A Tier 2 intervention must have an explicit educational purpose, such as curriculum-related learning, development of movement competence, knowledge, participation, self-regulation or personal and social learning through movement. In addition, it must either be formally scheduled or documented within the hospital-school program, or involve a teacher, PE professional or other educational staff member in planning, delivery or assessment. Frequency, intensity, duration, group size and mode of delivery will not determine eligibility.

Therapeutic exercise, physiotherapy, rehabilitation and active play will be included in Tier 2 only when they meet the educational decision rule in Table 1. Programs delivered solely as medical treatment, symptom management or recreation without an explicit educational objective will be excluded from Tier 2 but may be represented in Tier 1 through an eligible systematic review. Borderline cases will be independently assessed by two reviewers using the decision rule; study authors will be contacted when the educational purpose or organisational linkage cannot be determined from the report.

**Comparator**: A comparator will not be required because qualitative studies, single-group evaluations and descriptive designs are expected. When available, comparators such as usual care, non-PE educational activities, alternative movement programs or pre-intervention measurements will be extracted and considered in design-specific syntheses.

**Outcomes**: Priority outcomes will be organised into two complementary synthesis strands. The implementation/process strand will prioritise (i) safety and feasibility/implementation, including adverse events, and (ii) educational participation or engagement; these findings will be synthesised using quantitative descriptive, qualitative and mixed-method evidence, as appropriate. The effectiveness/outcome strand will prioritise (iii) physical functioning or motor competence and (iv) psychosocial well-being or health-related quality of life; these domains will be prioritised for comparative quantitative synthesis and GRADE assessment when the prespecified criteria for quantitative synthesis are met. Comparative evidence concerning safety or educational participation may also undergo quantitative synthesis and GRADE assessment when a sufficiently coherent body of evidence is available. Other clinical, cognitive, academic and implementation outcomes will be mapped and synthesised narratively.

**Study Designs**: Tier 1 will include systematic reviews and meta-analyses with an explicit search strategy, eligibility criteria and reproducible selection process. Narrative, scoping or integrative reviews will be excluded unless they meet these minimum systematic-review criteria. Tier 2 will include randomised trials, quasi-experimental and other non-randomised intervention studies, cohort, case-control and cross-sectional studies, qualitative studies, mixed-methods studies, and informative case reports or series. Protocols, editorials, commentaries and conference abstracts without sufficient outcome or implementation data will be excluded.

No restrictions will be applied by country, language, publication year, medical condition or length of stay. Contextual information, including the type and governance of the hospital school, educational staffing, clinical collaboration, resources and transition arrangements, will be extracted because these features may influence feasibility and transferability ( Pesce et al., 2021; Ciaccioni et al., 2025).

### 2.3 Data sources and search strategy

Separate search strategies will be developed for the umbrella review, the primary-study systematic review and the policy framework analysis because the components address different questions and evidence sources. Table 2 summarises the operational structure; complete line-by-line strategies for each database and operational procedures for policy framework analysis are reported as extended data ( Ciaccioni et al., 2026a).

**Table 2.** Component-specific search framework.

Component	Operational search structure
Tier 1: umbrella review	Sources: MEDLINE, Embase, CINAHL, PsycINFO, Scopus, Web of Science, SPORTDiscus and the Cochrane Database of Systematic Reviews. Concepts: child or adolescent AND hospital or inpatient AND physical activity, exercise, sport, movement or rehabilitation AND systematic-review terms. Hospital-school terminology will not be mandatory.
Tier 2: primary-study review	Sources: MEDLINE, Embase, CINAHL, PsycINFO, Scopus, Web of Science, SPORTDiscus, ERIC, Education Source and CENTRAL. Concepts: hospital school or in-hospital education AND physical education, physical activity, exercise, movement, sport or active play AND child or adolescent. No study-design filter will be applied.
Policy framework analysis	Sources: official international, European, national and regional organisation websites; ministries of education and health; legal databases; curriculum authorities; Youth Wiki; and recognised hospital-school networks. Concepts will combine hospital schooling or education during illness with PE, physical activity, movement, sport, curriculum, law, regulation, strategy, guideline and implementation terms in English and relevant local languages.

*Note*. CENTRAL: Cochrane Central Register of Controlled Trials; CINAHL: Cumulative Index to Nursing and Allied Health Literature; Embase: Excerpta Medica Database; ERIC: Education Resources Information Center; MEDLINE: Medical Literature Analysis and Retrieval System Online; PE: Physical Education.

• Tier 1 strategy. Searches will not include hospital-school terms as a mandatory concept because the purpose is to capture the broader hospital-based evidence. Reviews will be screened against the methodological and population criteria stated above, and the reference lists and citing literature of included reviews will be examined.
• Tier 2 strategy. Search sensitivity will be prioritised because hospital-school PE may be described using educational, clinical or recreational terminology. Broad movement terms will therefore be retained at the search stage, while the educational decision rule in Table 1 will be applied during full-text screening.
• Policy strategy and documentation. A common country-search template will specify the official domains searched, English and local-language terms, document categories, dates searched, result counts and reasons for exclusion. Search strings will combine hospital schooling or education during illness with PE, physical activity, movement, sport, curriculum and implementation terms. Where website search functions are inadequate, targeted web searches restricted to official domains will be used. Superseded documents will be retained only when needed to explain policy development and will be clearly labelled as historical.

Supplementary identification methods will include backward and forward citation searching, searches of relevant repositories and doctoral theses, and consultation with hospital educators, researchers and policy stakeholders. All expert contacts will receive the same written request. The search log will record the expert’s role, country, date of contact and nominated documents. A nominated document will be included only after independent eligibility verification and source authentication.

No date or language restrictions will be applied. Titles and abstracts in languages not spoken by the review team will be assessed using professional translation, institutional language support or verified machine translation as appropriate. Key eligibility, extraction and appraisal decisions will be checked by a reviewer able to assess the original language whenever feasible.

Database records will be exported to MS Excel (Microsoft 365), deduplicated and assigned to the relevant component. Searches will be rerun before final synthesis. The final reports will provide the complete strategies, dates, platforms, filters, deduplication procedures and any deviations from this protocol.

### 2.4 Use of artificial intelligence and automation tools

Any automation or artificial intelligence functionality will be used, if at all, only for supportive tasks such as deduplication, record organisation, translation support or citation prioritisation ( Thomas et al., 2026). Automated outputs will not determine eligibility, extraction, appraisal, coding, theme development, certainty ratings or interpretation. Reviewers will verify all outputs and retain direct responsibility for every methodological decision. The tool name, developer, version, purpose, date of use, input type, verification procedure and any detected errors will be documented in the final report.

### 2.5 Screening and study selection

The reviewer workflow is summarised in Table 3 and will apply separately to Tier 1, Tier 2 and the policy component. Before formal screening, reviewers will pilot the eligibility criteria on a shared sample and refine the guidance without altering the registered review questions. Titles and abstracts, followed by full texts, will be assessed independently by two reviewers. Disagreements will be resolved through discussion and, when necessary, adjudication by a third reviewer. Full-text exclusions and reasons will be recorded, and selection will be reported using component-specific PRISMA flow diagrams. Authors will be contacted when essential eligibility information is unclear.

**Table 3.** Reviewer workflow across the three review components.

Stage	Reviewer process	Resolution and documentation
Search validation	A second reviewer or information-search lead will check each strategy against the relevant eligibility criteria before execution.	Search versions, dates, platforms and amendments will be retained in the search log.
Title, abstract and full-text screening	Two reviewers will assess records independently after a shared calibration exercise.	Disagreements will be resolved by consensus or third-reviewer adjudication; full-text exclusion reasons will be recorded.
Data extraction	One reviewer will extract data and a second reviewer will verify all fields against the source.	Discrepancies and author contacts will be documented.
Risk of bias and critical appraisal	Two reviewers will appraise each included unit independently using the tool appropriate to its design or document type.	Consensus or third-reviewer adjudication; domain-level judgments will be retained.
Qualitative synthesis	Two reviewers will independently code a diverse calibration sample; one will code the remaining findings and the second will verify coding and themes.	A codebook, reflexive notes, divergent cases and theme revisions will form an audit trail.
Certainty assessment	Two reviewers will independently prepare GRADE or GRADE-CERQual judgments for predefined priority findings.	Consensus or third-reviewer adjudication; reasons for judgments will be reported.
Policy-document analysis	Two reviewers will independently confirm eligibility; one will extract and a second will verify.	Expert nominations will be logged and subjected to the same eligibility and authenticity checks as documents found through standard searches.

*Note*. GRADE: Grading of Recommendations Assessment, Development and Evaluation; CERQual: Confidence in the Evidence from Reviews of Qualitative research.

### 2.6 Data extraction

Component-specific extraction forms will be developed, piloted and accompanied by a coding manual ( Khudair et al., 2022; Lee et al., 2025a; Lee et al., 2025b). For Tier 2, extracted items will include publication and design, country and hospital-school context, participant characteristics and diagnoses, educational purpose, intervention content and dose, delivery personnel and professional preparation, curriculum linkage, comparator, outcomes and instruments, time points, numerical or qualitative findings, adverse events, implementation processes, barriers and facilitators, funding and conflicts of interest.

For Tier 1, extraction will cover review objectives, databases and search dates, eligibility criteria, included study designs and populations, intervention categories, outcomes, synthesis methods, effect estimates, adverse events, implementation findings, certainty assessments and authors’ conclusions. Primary-study overlap across included reviews will be mapped and quantified using the Corrected Covered Area ( Higgins et al., 2023). No new meta-analysis will combine pooled estimates from overlapping reviews.

For policy documents, extraction will record the document title, issuing authority, jurisdiction, legal or administrative status, date, current or superseded status, target population, references to PE or related movement provision, curricular expectations, professional roles, safety and adaptation requirements, funding or resource provisions, implementation and monitoring mechanisms, and links between education and health authorities. Direct quotations will be limited to short excerpts required to support interpretation.

One reviewer will extract each record and a second reviewer will verify all fields against the source. Discrepancies will be resolved according to Table 3. When multiple reports describe the same study or policy instrument, they will be linked and treated as one unit of evidence. Authors or issuing bodies may be contacted for missing information, but unverified information will not replace the published or official record.

### 2.7 Risk of bias and quality assessment

Critical appraisal will be selected according to evidence type and will be completed independently by two reviewers as specified in Table 3. Appraisal results will inform synthesis, certainty assessment and interpretation but will not be used as an automatic exclusion threshold.

Randomised trials will be assessed with Cochrane Risk of Bias 2 (RoB 2), and non-randomised intervention studies with Risk Of Bias In Non-randomized Studies of Interventions (ROBINS-I) ( Sterne et al., 2016; Sterne et al., 2019). Observational and case-based quantitative evidence will be assessed using the Joanna Briggs Institute (JBI) critical appraisal instrument appropriate to the reported design ( https://jbi.global/critical-appraisal-tools). Mixed-methods studies will be appraised through the Mixed Methods Appraisal Tool-MMAT ( Hong et al., 2018).

Qualitative studies will be appraised with the Critical Appraisal Skills Programme checklist, with attention to the appropriateness of the design, recruitment, data collection, reflexivity, analysis, ethical considerations and coherence between data and interpretation ( Long et al., 2020).

Systematic reviews included in Tier 1 will be appraised using AMSTAR 2 ( Shea et al., 2017). The review team will also examine the recency and scope of each review, the risk of bias of its included studies, and overlap between reviews. Conclusions from critically low-quality reviews will not be treated as equivalent to conclusions from reviews with stronger methods.

Policy and regulatory documents will be appraised according to document type. AGREE II will be used only for documents that meet the characteristics of a practice or implementation guideline ( Brouwers et al., 2010). Policy texts will be assessed using the JBI approach for textual policy evidence, including credibility, authority, purpose, evidence base and transparency of development ( McArthur et al., 2025). Grey-literature source characteristics will additionally be documented using the Authority, Accuracy, Coverage, Objectivity, Date, Significance (AACODS) Checklist ( Seniutis et al., 2026; Tyndall, 2010). For laws and regulations, their issuing authority, legal force, currency, scope, accessibility and implementation provisions will be recorded.

Appraisal judgments will be presented by domain; sensitivity analyses or structured comparisons will examine whether conclusions differ when evidence at high or critical risk of bias is omitted, provided sufficient studies are available.

### 2.8 Data synthesis and analysis

Narrative synthesis will be the primary approach for both review tiers because heterogeneity is expected in settings, populations, educational purposes, interventions, designs and outcomes. Tier 1 findings will be summarised by review scope and quality, with explicit attention to primary-study overlap and transferability to hospital-school PE. For Tier 2, synthesis will distinguish implementation/process evidence from effectiveness/outcome evidence. Safety, feasibility, implementation and educational participation or engagement will primarily be integrated through narrative, qualitative and, where appropriate, mixed-method synthesis. Physical functioning or motor competence and psychosocial well-being or health-related quality of life will be prioritised for comparative quantitative synthesis. This distinction is analytical rather than absolute, as sufficiently comparable comparative data on safety or educational participation may also support quantitative synthesis. Policy documents will first be synthesised by jurisdiction, document type, legal status and substantive provision, followed by cross-jurisdictional comparison.

Meta-analysis will be considered only when at least three independent studies are sufficiently comparable in population, intervention purpose and content, design, comparator, outcome construct and assessment time point, and when effect estimates can be derived without double-counting participants. Randomised and non-randomised studies will not be pooled together. Random-effects models will be used, with risk ratios or odds ratios for dichotomous outcomes and mean differences or standardised mean differences for continuous outcomes, each with 95% confidence intervals. Statistical heterogeneity will be examined using I-squared and tau-squared, but pooling decisions will be based primarily on clinical, educational and methodological comparability. Subgroup and sensitivity analyses will be undertaken only when adequately supported and will prioritise age group, clinical condition, intervention type, delivery professional and risk of bias. Publication-bias analyses will be considered only when the number of studies is sufficient for meaningful interpretation ( Higgins et al., 2023; Borenstein et al., 2021).

Qualitative findings will be synthesised using the three-stage thematic-synthesis process of line-by-line coding, development of descriptive themes and generation of analytical themes ( Thomas and Harden, 2008; Green and Thorogood, 2018). Two reviewers will independently code an initial sample representing different settings and participant perspectives, compare interpretations and agree a provisional codebook. One reviewer will then code the remaining findings and the second will verify coding and theme development. Reflexive notes, coding decisions, divergent cases and changes to the codebook will be retained in an audit trail. Participant quotations and study-author interpretations will be distinguished. Policy and empirical syntheses will subsequently be integrated in a matrix examining convergence, divergence, implementation conditions, transferability and unresolved gaps.

### 2.9 Certainty of evidence assessment

GRADE will be applied to sufficiently coherent bodies of comparative quantitative evidence, with physical functioning or motor competence and psychosocial well-being or health-related quality of life designated as the primary domains for quantitative certainty assessment. Safety/adverse events and educational participation or engagement will also be assessed using GRADE when they are reported as sufficiently comparable comparative outcomes; otherwise, they will contribute primarily to the implementation/process synthesis. Separate certainty judgments will be made by study design and outcome, considering risk of bias, inconsistency, indirectness, imprecision and publication bias, with upgrading factors applied where appropriate. GRADE-CERQual (Confidence in the Evidence from Reviews of Qualitative research) will be applied to a limited set of priority qualitative findings concerning safety, acceptability, barriers, facilitators, educational participation and engagement, and stakeholder experience, considering methodological limitations, coherence, adequacy and relevance ( Lewin et al., 2018; Brozek et al., 2021; Kolovelonis et al., 2024). Certainty will not be assigned to isolated case reports, purely descriptive mappings or legal obligations. Summary-of-findings tables will report the rationale for each judgment and distinguish evidence from Tier 1, Tier 2 and the policy analysis.

## 3. Discussion

This protocol addresses an under-synthesised area at the intersection of education, health and physical activity. Its central methodological challenge is that PE, clinical exercise, rehabilitation and active play may coexist in hospital settings while serving different purposes ( Issaka and Hopkins, 2015; West et al., 2019; Cistulli et al., 2025) with operational definitions and decision rules intended to preserve the educational focus of Tier 2 while allowing Tier 1 to provide a broader clinical and implementation context.

The multi-component design may clarify how PE has been conceptualised and delivered, which outcomes have been assessed, and which implementation conditions have been reported. However, the review will not assume that hospital-based physical activity programs are educationally equivalent to PE or that evidence from one diagnosis, profession or healthcare system is transferable to another ( Reid, 2010; Braam et al., 2016; Santos et al., 2020; Crowder et al., 2022). Transferability will be examined explicitly with reference to educational purpose, clinical constraints, staffing and governance.

The policy analysis may help explain whether variation in provision is associated with the presence or absence of curriculum requirements, professional responsibilities, safety guidance, intersectoral coordination or implementation resources. Its interpretation will distinguish legal obligations from recommendations and practice guidelines. The analysis is intended to identify policy patterns and gaps rather than rank countries or infer implementation solely from the existence of a document ( European Union, 2008; European Commission, 2023; Amornsriwatanakul et al., 2025).

Several limitations are anticipated. The empirical evidence may be sparse, heterogeneous and at risk of bias, which may preclude quantitative pooling and result in low-certainty conclusions. Terminology may be inconsistent, relevant educational activities may be reported in clinical literature, and policy documents may be difficult to locate, translate or compare across legal systems. Standardised searches, transparent decision rules, duplicate screening, document-type-specific appraisal and an audit trail are intended to reduce, but cannot eliminate, these limitations. Expert consultation may improve document identification, although it may introduce unequal coverage across jurisdictions; expert nominations will therefore remain supplementary to the standardised search and will undergo the same eligibility checks ( Browne et al., 2019; Brozek et al., 2021; Higgins et al., 2023).

The completed reviews are intended to provide a transparent account of the available evidence and policy landscape. Depending on the findings and their certainty, they may inform subsequent stakeholder consultation, research priorities and the development of context-sensitive guidance for PE in hospital schools. They will not, by themselves, establish universal implementation standards ( Kohl III et al., 2013; Longmuir et al., 2013; American Public Health Association, 2021; World Health Organization, 2010).

## Ethics and dissemination

This multi-tiered review (encompassing a systematic review, umbrella review, and policy framework analysis) will rely exclusively on secondary data from published studies and publicly available policy documents. No primary data collection involving human participants will be conducted, and thus no formal ethics approval is required.

The results will be disseminated in peer-reviewed publications, conference presentations, open repositories and targeted materials for hospital-school educators, healthcare professionals and policy stakeholders. Dissemination products will distinguish review findings from subsequent recommendations and will report uncertainty, contextual limitations and evidence gaps.

## Data Availability

The manuscript contains the following Extended Data: Ciaccioni S, Pesce C, Benzing V, Schmidt M, Magalhães P, Rosário P, Pedrosa Leal L, Pontes CM, Oddi L, Basciano M, Betello F, Bradic S & Capranica L: Extended data for “MSCA HOPE - Physical education in hospital schools: A protocol paper for systematic and umbrella reviews with policy framework analysis”. *Zenodo*; 2026. https://doi.org/10.5281/zenodo.21825405. Data are available under the terms of the Creative Commons Zero “No rights reserved” data waiver ( CC0 1.0 Public domain dedication).

## References

[ref1] American Public Health Association: *Supporting Physical Education in Schools for All Youth.* Washington, DC, USA: American Public Health Association;2021.

[ref2] Amornsriwatanakul A Chinapong S Wattanapisit A : Thailand National Physical Activity Promotional and Action Plans for Children and Youth: Development and Implementation Challenges. *J Phys Act Health.* 2025;22:1453–1463. 10.1123/jpah.2025-0083 40846306

[ref3] Borenstein M Hedges LV Higgins JP : *Introduction to meta-analysis.* John Wiley & Sons;2021. 10.1002/9781119558378

[ref4] Bowen GA : Document analysis as a qualitative research method. *Qualitative research journal.* 2009;9:27–40. 10.3316/QRJ0902027

[ref5] Braam KI Van Der Torre P Takken T : Physical exercise training interventions for children and young adults during and after treatment for childhood cancer. *Cochrane Database Syst Rev.* 2016;2017:CD008796. 10.1002/14651858.CD008796.pub3 PMC6464400 27030386

[ref6] Brouwers MC Kho ME Browman GP : For the AGREE Next Steps Consortium. AGREE II: Advancing guideline development, reporting and evaluation in healthcare. *Canadian Medical Association Journal.* 2010;182:E839–E842. 10.1503/cmaj.090449 20603348 PMC3001530

[ref7] Brown P Brunnhuber K Chalkidou K : How to formulate research recommendations. *BMJ.* 2006;333:804–806. 10.1136/bmj.38987.492014.94 17038740 PMC1602035

[ref8] Browne J Coffey B Cook K : A guide to policy analysis as a research method. *Health Promotion International.* 2019;34:1032–1044. 10.1093/heapro/day052 30101276

[ref9] Brozek JL Canelo-Aybar C Akl EA : GRADE Guidelines 30: the GRADE approach to assessing the certainty of modeled evidence-An overview in the context of health decision-making. *J Clin Epidemiol.* 2021;129:138–150. 10.1016/j.jclinepi.2020.09.018 32980429 PMC8514123

[ref10] Bull FC Al-Ansari SS Biddle S : World Health Organization 2020 guidelines on physical activity and sedentary behaviour. *Br J Sports Med.* 2020;54:1451–1462. 10.1136/bjsports-2020-102955 33239350 PMC7719906

[ref11] Catenazzo T : La scuola in ospedale e l’istruzione domiciliare: formazione degli insegnanti e linee di indirizzo nazionali [in Italian]. *Carocci.* 2019.

[ref12] Catenazzo T : *Quando la scuola è di casa: Prassi inclusive ed educazione di prossimità nel lavoro del docente ospedaliero e domiciliare [in Italian].* Mimesis;2024.

[ref13] Ciaccioni S Compernolle S Lerfald M : Modifiable determinants of older adults’ physical activity and sedentary behavior in community and healthcare settings: a DE-PASS systematic review and meta-analysis. *Eur Rev Aging Phys Act.* 2025;22:9. 10.1186/s11556-025-00373-y 40413376 PMC12103017

[ref14] Ciaccioni S Oddi L Kolovelonis A : Modifiable determinants of adolescents’ physical activity and sedentary behaviour in school, home and mixed settings: A DE-PASS systematic review and meta-analysis of self-reported data with a holistic analysis. *Mental Health and Physical Activity.* 2026a;31:100803–100820. 10.1016/j.mhpa.2026.100803

[ref15] Ciaccioni S Pesce C Benzing V : Extended data for “MSCA HOPE - Physical education in hospital schools: A protocol paper for systematic and umbrella reviews with policy framework analysis”. *Zenodo.* 2026b. 10.5281/zenodo.21825405

[ref16] Cistulli M Bellucci M Casella R : Educazione fisica nella scuola in ospedale *Percorsi didattici ventennali [In Italian]*. *Calzetti & Mariucci Ed.* 2025.

[ref17] Commission E Education E Agency CE : *Physical education and sport at school in Europe.* Publications Office of the European Union;2013.

[ref18] Commission E Education E Agency CE : *The situation of young people in the European Union – EU youth report 2024.* Publications Office of the European Union;2025.

[ref19] Crowder SL Buro AW Stern M : Physical activity interventions in pediatric, adolescent, and young adult cancer survivors: a systematic review. *Support Care Cancer.* 2022;30:4635–4649. 10.1007/s00520-022-06854-5 35064822 PMC9175508

[ref20] Davies D Hartfield D Wren T : Children who ‘grow up’ in hospital: Inpatient stays of six months or longer. *Paediatr Child Health.* 2014;19:533–536. 10.1093/pch/19.10.533 25587232 PMC4276387

[ref21] Dixon CE : *Teaching Physical Education in Nontraditional Settings [Doctoral Thesis].* Auburn University;2020.

[ref22] European Commission: *2024 Annual Work Programme “Erasmus+”: the Union Programme for Education, Training.* Youth and Sport. European Commission;2023.

[ref23] European Parliament : *Directorate-General for Internal Policies, Policy Department A: The fight against cancer is a team sport: the role of education and sport.* Brussels: European Parliament;2016. 10.2861/29500

[ref24] European Union : *Guidelines recommended policy actions in support of health-enhancing physical activity.* EU Working Group “Sport & Health”;2008.

[ref25] GloPL Consortium: *Quadro d'azione globale per l’alfabetizzazione motoria - traduzione italiana [The Global Physical Literacy Action Framework].* Geelong, VIC, Australia: Consorzio GloPL;2026. 10.26187/q0zy-4158

[ref26] Green J Thorogood N : *Qualitative methods for health research.* 2018. 10.4135/9781036234386

[ref27] Gutenbrunner C Meyer T Melvin J : Towards a conceptual description of Physical and Rehabilitation Medicine. *J Rehabil Med.* 2011;43(9):760–764. 10.2340/16501977-0866 21874211

[ref28] Higgins J Thomas J Chandler J : *Cochrane handbook for systematic reviews of interventions version 6.4.* Cochrane;2023. [Accessed]. Reference Source

[ref46] Hong QN Fàbregues S Bartlett G : The Mixed Methods Appraisal Tool (MMAT) version 2018 for information professionals and researchers. *Education for information.* 2018;34(4):285–291. 10.3233/EFI-180221

[ref29] Issaka A Hopkins L : Introducing physical education to hospital learning–can patients participate? *Asia-Pacific Journal of Health, Sport and Physical Education.* 2015;6:23–40. 10.1080/18377122.2014.997859

[ref30] Jiliberto F Zarate Alva N : Influencing Factors In-Hospital School Education: Exploring the Context From the Teacher's Perspective. *Contin Educ.* 2024;6:1–21. 10.5334/cie.126 39897589 PMC11784520

[ref31] Khudair M Marcuzzi A Ng K : DE-PASS Best Evidence Statement (BESt): modifiable determinants of physical activity and sedentary behaviour in children and adolescents aged 5-19 years-a protocol for systematic review and meta-analysis. *BMJ Open.* 2022;12:e059202. 10.1136/bmjopen-2021-059202 36127107 PMC9490573

[ref32] Kohl HW III Cook HD Activity COP : The Effectiveness of Physical Activity and Physical Education Policies and Programs: Summary of the Evidence. *Educating the Student Body: Taking Physical Activity and Physical Education to School.* National Academies Press (US);2013. 24851299

[ref33] Kolovelonis A Syrmpas I Marcuzzi A : DE-PASS best evidence statement (BESt): determinants of adolescents’ device-based physical activity and sedentary behaviour in settings: a systematic review and meta-analysis. *BMC Public Health.* 2024;24:1706. 10.1186/s12889-024-19136-y 38926707 PMC11202347

[ref34] Lee Y Capranica L Pesce C : Olympic combat sports and mental health in children and adolescents with disability: A protocol paper for systematic review. *PLoS One.* 2025a;20(2):e0301949. 10.1371/journal.pone.0301949 39928614 PMC11809909

[ref35] Lee Y Guidotti F Capranica L : Olympic combat sports and mental health in children and adolescents with disability: A systematic review of controlled trials. *Front Psychol.* 2025b;16:1567978. 10.3389/fpsyg.2025.1567978 40376492 PMC12078338

[ref36] Lewin S Booth A Glenton C : Applying GRADE-CERQual to qualitative evidence synthesis findings: introduction to the series. *Implement Sci.* 2018;13(2). 10.1186/s13012-017-0688-3 29384079 PMC5791040

[ref37] Ling FCM Khudair M Ng K : DE-PASS Best Evidence Statement (BESt): Determinants of self-report physical activity and sedentary behaviours in children in settings: A systematic review and meta-analyses. *PLoS One.* 2024;19:e0309890. 10.1371/journal.pone.0309890 39585854 PMC11588252

[ref38] Long HA French DP Brooks JM : Optimising the value of the critical appraisal skills programme (CASP) tool for quality appraisal in qualitative evidence synthesis. *Research Methods in Medicine & Health Sciences.* 2020;1:31–42. 10.1177/2632084320947559

[ref39] Longmuir PE Brothers JA De Ferranti SD : Promotion of physical activity for children and adults with congenital heart disease: a scientific statement from the American Heart Association. *Circulation.* 2013;127:2147–2159. 10.1161/CIR.0b013e318293688f 23630128

[ref40] Ludgério MJB Pontes CM Dos Santos BLC : Pedagogical practices developed with children through hospital classes: An integrative literature review. *Journal of Pediatric Nursing-Nursing Care of Children & Families.* 2023;72:E10–E18. 10.1016/j.pedn.2023.05.014 37302968

[ref41] Martins J Costa J Sarmento H : Adolescents’ Perspectives on the Barriers and Facilitators of Physical Activity: An Updated Systematic Review of Qualitative Studies. *Int J Environ Res Public Health.* 2021;18:4954. 10.3390/ijerph18094954 34066596 PMC8125166

[ref42] Marusak HA Iadipaolo AS Cohen C : Martial Arts-Based Therapy Reduces Pain and Distress Among Children with Chronic Health Conditions and Their Siblings. *Journal of Pain Research.* 2020;13:3467–3478. 10.2147/JPR.S283364 33402843 PMC7778380

[ref43] Mclennan N Thompson J : *Quality physical education (QPE): Guidelines for policy makers.* Unesco Publishing;2015.

[ref44] Metzler MW Mckenzie TL Van Der Mars H : Health optimizing physical education (HOPE): A new curriculum for school programs—Part 2: Teacher knowledge and collaboration. *Journal of Physical Education, Recreation & Dance.* 2013;84:25–34. 10.1080/07303084.2013.779531

[ref45] McArthur A Cooper A Edwards D : Textual evidence systematic reviews series paper 3: critical appraisal of evidence from narrative, opinion, and policy. *JBI Evidence Synthesis.* 2025;23(5):833–839. 10.11124/JBIES-24-00293 39905824

[ref47] Opstoel K Chapelle L Prins FJ : Personal and social development in physical education and sports: A review study. *European Physical Education Review.* 2020;26(4):797–813. 10.1177/1356336X19882054

[ref48] Pagnani B Caldoro S Guidi A : *Scuola in ospedale. Una formazione di qualità per integrare benessere-apprendimento-salute [In Italian].* Firenze: Le Monnier;2004.

[ref49] Pellegrini AD Smith PK : Physical activity play: The nature and function of a neglected aspect of playing. *Child Dev.* 1998;69(3):577–598. 10.1111/j.1467-8624.1998.tb06226.x 9680672

[ref50] Pesce C Vazou S Benzing V : Effects of chronic physical activity on cognition across the lifespan: a systematic meta-review of randomized controlled trials and realist synthesis of contextualized mechanisms. *Int Rev Sport Exerc Psychol.* 2021;16(1):722–760. 10.1080/1750984x.2021.1929404

[ref51] Reid TR : *The healing of America: A global quest for better, cheaper, and fairer health care.* Penguin;2010.

[ref52] Santos SDS Moussalle LD Heinzmann-Filho JP : Effects of Physical Exercise during Hospitalization in Children and Adolescents with Cancer: A Systematic Review. *Rev Paul Pediatr.* 2020;39:e2019313. 10.1590/1984-0462/2021/39/2019313 33027320 PMC7537404

[ref53] Seniutis M Sas A Gružauskas V : Exploring more-than-human ethics of large language models in social service delivery. *Ethics and Social Welfare.* 2026;20:317–338. 10.1080/17496535.2025.2593823

[ref54] Shamseer L Moher D Clarke M : Preferred reporting items for systematic review and meta-analysis protocols (PRISMA-P) 2015: elaboration and explanation. *BMJ.* 2015;349:g7647. 10.1136/bmj.g7647 25555855

[ref55] Shea BJ Reeves BC Wells G : AMSTAR 2: a critical appraisal tool for systematic reviews that include randomised or non-randomised studies of healthcare interventions, or both. *BMJ.* 2017;358:j4008. 10.1136/bmj.j4008 28935701 PMC5833365

[ref56] Shields N Synnot AJ Barr M : Perceived barriers and facilitators to physical activity for children with disability: a systematic review. *British Journal of Sports Medicine.* 2012;46:989–997. 10.1136/bjsports-2011-090236 21948121

[ref57] Sterne JA Hernan MA Reeves BC : ROBINS-I: a tool for assessing risk of bias in non-randomised studies of interventions. *BMJ.* 2016;355:i4919. 10.1136/bmj.i4919 27733354 PMC5062054

[ref58] Sterne JC Savovic J Page MJ : RoB 2: a revised tool for assessing risk of bias in randomised trials. *BMJ.* 2019;366:l4898. 10.1136/bmj.l4898 31462531

[ref59] Taylor NF Dodd KJ Shields N : Therapeutic exercise in physiotherapy practice is beneficial: a summary of systematic reviews 2002–2005. *Australian Journal of Physiotherapy.* 2007;53(1):7–16. 10.1016/S0004-9514(07)70057-0 17326734

[ref60] Tenenbaum G Driscoll MP : *Methods of research in sport sciences: Quantitative and qualitative approaches.* Meyer & Meyer Verlag;2005.

[ref61] Theobald U Mayer J Giese M : Physical education for students with chronic health conditions: A scoping review. *Empirische Pädagogik.* 2025;39(3):228–247. 10.62350/QEEG6790

[ref62] Thomas J Hair K Noel-Storr A : Responsible use of AI in Evidence Synthesis (RAISE 2026): building and evaluating AI evidence synthesis tools (version 3; updated 13 March 2026). 2026.

[ref63] Thomas J Harden A : Methods for the thematic synthesis of qualitative research in systematic reviews. *BMC Med Res Methodol.* 2008;8:45. 10.1186/1471-2288-8-45 18616818 PMC2478656

[ref64] Tyndall J : *AACODS Checklist.* Flinders University;2010. https://dspace.flinders.edu.au/

[ref65] UNESCO: International charter of physical education, physical activity and sport. 2015.

[ref66] West SL Banks L Schneiderman JE : Physical activity for children with chronic disease; a narrative review and practical applications. *BMC Pediatr.* 2019;19:12. 10.1186/s12887-018-1377-3 30621667 PMC6325687

[ref67] World Health Organization: Global recommendations on physical activity for health. 2010. 26180873

[ref68] World Health Organization: Global status report on physical activity 2022: web annex: global action plan on physical activity monitoring framework, indicators and data dictionary. *Global status report on physical activity 2022: web annex: global action plan on physical activity monitoring framework, indicators and data dictionary.* 2022.

