# Supplementary material for: MSCA HOPE - Physical education in hospital schools: A protocol paper for systematic and umbrella reviews with policy framework analysis
Source: Open Res Eur. 2026 Aug 27;6:188. Originally published 2026 Jun 1. [Version 2] doi: 10.12688/openreseurope.23993.2 (PMC13562943; doi:10.12688/openreseurope.23993.2)
Supplement: Peer review history

# Reviewer report: Reviewer response for version 2

**DOI:** 10.21956/openreseurope.27048.r79786

**Published:** 2026-09-09

**Authors:** Francesca Greco

**Affiliations:** 1 Department of Experimental and Clinical Medicine, University “Magna Græcia” of Catanzaro, Catanzaro, Italy

Authors fully addressed my previous comments.

Is the study design appropriate for the research question?

Yes

Are sufficient details of the methods provided to allow replication by others?

Partly

Is the rationale for, and objectives of, the study clearly described?

Yes

Are the datasets clearly presented in a useable and accessible format?

Yes

Reviewer Expertise:

Exercise science researcher

I confirm that I have read this submission and believe that I have an appropriate level of expertise to confirm that it is of an acceptable scientific standard.

# Reviewer report: Reviewer response for version 2

**DOI:** 10.21956/openreseurope.27048.r79785

**Published:** 2026-09-04

**Authors:** Federico Roggio

**Affiliations:** 1 Department of Biomedical and Biotechnological Sciences, Section of Anatomy, Histology and Movement Science, School of Medicine,, University of Catania, Catania, Italy

The authors have adequately addressed the comments raised in my previous review. In particular, the revised manuscript now provides clearer operational definitions and eligibility criteria for physical education, a more reproducible component-specific search strategy, a proportionate synthesis plan, a substantially strengthened policy framework analysis, and clearer procedures for qualitative synthesis and reviewer responsibilities. The revised wording is also more consistent with the prospective nature of a protocol paper. I consider my previous concerns satisfactorily resolved and have no further substantive comments.

Is the study design appropriate for the research question?

Yes

Are sufficient details of the methods provided to allow replication by others?

Partly

Is the rationale for, and objectives of, the study clearly described?

Partly

Are the datasets clearly presented in a useable and accessible format?

No

Reviewer Expertise:

Public health - human movement sciences

I confirm that I have read this submission and believe that I have an appropriate level of expertise to confirm that it is of an acceptable scientific standard.

# Reviewer report: Reviewer response for version 2

**DOI:** 10.21956/openreseurope.27048.r79784

**Published:** 2026-08-31

**Authors:** Henri Tilga

**Affiliations:** 1 Institute of Sport Sciences and Physiotherapy, Faculty of Medicine, University of Tartu, Tartu, Estonia

Authors have done well in revising their manuscript.

Is the study design appropriate for the research question?

Yes

Are sufficient details of the methods provided to allow replication by others?

Yes

Is the rationale for, and objectives of, the study clearly described?

Yes

Are the datasets clearly presented in a useable and accessible format?

Yes

Reviewer Expertise:

Physical education

I confirm that I have read this submission and believe that I have an appropriate level of expertise to confirm that it is of an acceptable scientific standard.

# Reviewer report: Reviewer response for version 1

**DOI:** 10.21956/openreseurope.25974.r76193

**Published:** 2026-07-29

**Authors:** Francesca Greco

**Affiliations:** 1 Department of Experimental and Clinical Medicine, University “Magna Græcia” of Catanzaro, Catanzaro, Italy

Thank you for the opportunity to evaluate this manuscript. This protocol addresses an important and insufficiently explored area that lies at the intersection of hospital education, paediatric health, and educational policy. 

 Overall, the protocol is well conceived and addresses a relevant research question. However, some methodological aspects would benefit from further refinement. Please find my suggestions below:

 1- Please better define the range of concepts used in the protocol. Specifically, it would be beneficial to better elucidate the meaning of physical education. It could nbe useful to include a decision framework for study selection.

 2 - The criteria for undertaking quantitative 

 3 - The policy analysis represents one of the protocol's most innovative components but currently lacks sufficient methodological detail. The authors should clearly define the types of policy documents that will be considered eligible and explain the procedures that will be used to identify grey literature.

 4 -The wording throughout the manuscript should consistently distinguish between planned methods and future outcomes.

Is the study design appropriate for the research question?

Yes

Are sufficient details of the methods provided to allow replication by others?

Partly

Is the rationale for, and objectives of, the study clearly described?

Yes

Are the datasets clearly presented in a useable and accessible format?

Yes

Reviewer Expertise:

Exercise science researcher

I confirm that I have read this submission and believe that I have an appropriate level of expertise to confirm that it is of an acceptable scientific standard, however I have significant reservations, as outlined above.

# Response

**Published:** 2026-08-17

**Authors:** Simone Ciaccioni

Dear Dr Greco,

 We sincerely thank you for your positive assessment and constructive recommendations. Your comments helped us improve the conceptual clarity, methodological detail, reproducibility, and protocol-oriented wording of the manuscript. Our point-by-point responses are provided below.

**Comment 1. Definition of concepts and decision framework**

 Please better define the range of concepts used in the protocol. Specifically, it would be beneficial to better elucidate the meaning of physical education. It could be useful to include a decision framework for study selection.

**Response:**

 We agree and have substantially clarified the conceptual boundaries between physical education, physical activity, therapeutic exercise, rehabilitation, and active play. A new terminology and screening decision table, now presented as Table 1, defines each concept and specifies its eligibility for Tier 2. Physical education is operationally defined as planned movement-based learning with explicit educational aims related to curriculum, movement competence, knowledge, participation, self-regulation, or personal and social development. For inclusion in Tier 2, an intervention must have an explicit educational purpose and must also meet at least one of the following criteria:

1. formal integration into the hospital-school programme; or
2. documented involvement of educational staff in planning, delivery, or assessment.

Therapeutic exercise, physiotherapy, rehabilitation, and active play delivered solely for clinical treatment, symptom management, or recreation will be excluded from Tier 2, although they may be represented in Tier 1 through eligible systematic reviews. The new decision framework also addresses borderline interventions combining educational, therapeutic, and recreational purposes. These cases will undergo independent duplicate full-text assessment, and study authors will be contacted where the educational purpose or hospital-school connection cannot be determined from the published report.

**Comment 2. Criteria for quantitative synthesis**

 The reviewer’s second comment appears to be incomplete in the published report: “The criteria for undertaking quantitative...”. We interpreted this as a request to clarify the conditions under which quantitative synthesis or meta-analysis would be undertaken.

**Response:**

 We have revised the synthesis plan to identify narrative synthesis as the primary analytical approach. Meta-analysis will be considered only when at least three independent studies are sufficiently comparable in relation to:

- population;
- intervention purpose and content;
- study design;
- comparator;
- outcome construct; and
- assessment time point.

Randomised and non-randomised studies will not be pooled together, and quantitative synthesis will not be undertaken when it would result in double-counting participants. Decisions concerning pooling will be based primarily on clinical, educational, and methodological comparability rather than statistical heterogeneity alone. Case reports, case series, and descriptive evidence will be used for evidence mapping and narrative interpretation rather than pooled effectiveness estimates. We have also organised the predefined priority domains into two complementary synthesis strands. Safety and feasibility/implementation, together with educational participation or engagement, will primarily contribute to an implementation/process synthesis drawing on quantitative descriptive, qualitative and mixed-method evidence. Physical functioning or motor competence and psychosocial well-being or health-related quality of life will be prioritised for comparative quantitative synthesis and GRADE assessment. However, this distinction is not absolute: safety or educational participation outcomes will also be eligible for quantitative synthesis and GRADE when sufficiently comparable comparative evidence is available. This structure is intended to keep the synthesis proportionate to the anticipated evidence base while avoiding the application of the same analytical approach to conceptually different types of evidence.

**Comment 3. Policy analysis and grey literature**

 The policy analysis represents one of the protocol's most innovative components but currently lacks sufficient methodological detail. The authors should clearly define the types of policy documents that will be considered eligible and explain the procedures that will be used to identify grey literature.

**Response:**

 We agree and have expanded the policy-analysis methods throughout the manuscript and in a dedicated extended-data protocol. Eligible documents are now explicitly defined as binding and non-binding instruments, including:

- legislation;
- regulations;
- ministerial decrees and circulars;
- curriculum frameworks;
- national and regional strategies;
- official standards;
- implementation guidance; and
- international normative or recommendation documents.

Grey-literature searches will follow a standardised country-level procedure documenting the official domains searched, English and local-language terms, document categories, dates searched, result counts, and reasons for exclusion. Sources will include ministries of education and health, legal databases, curriculum authorities, official international and regional organisations, Youth Wiki, and recognised hospital-school networks. Where the internal search functions of official websites are inadequate, targeted web searches restricted to official domains will be used. Expert consultation will serve only as a supplementary method for identifying potentially missing documents. All experts will receive the same written request, and their role, country, date of contact, and nominated documents will be recorded. Each nominated document will undergo the same eligibility and source-authentication procedures as documents identified through the standard searches. The appraisal approach has also been differentiated by document type. AGREE II will be used only for practice or implementation guidelines. Other policy texts will be assessed using the JBI approach for textual policy evidence, supplemented by AACODS criteria for grey literature. Laws and regulations will be characterised according to issuing authority, legal force, currency, scope, accessibility, and implementation provisions.

**Comment 4. Distinction between planned methods and future outcomes**

 The wording throughout the manuscript should consistently distinguish between planned methods and future outcomes.

**Response:**

 We agree and have revised the wording throughout the Abstract, Introduction, Discussion, conclusion, and dissemination sections. Statements that appeared to anticipate the findings or impact of the future reviews have been replaced with more cautious protocol-oriented formulations. For example, the manuscript now states that the reviews are “intended to” clarify the evidence and policy landscape and “may inform” subsequent stakeholder consultation, research priorities, and guideline development. The revised Discussion also acknowledges that the available evidence may be sparse, heterogeneous, and at risk of bias and clarifies that the reviews will not, by themselves, establish universal implementation standards. This wording more clearly distinguishes planned procedures, possible findings, and potential future applications.

**Are sufficient details of the methods provided to allow replication by others?** **Original assessment: Partly**

**Response:**

 To improve reproducibility, we added operational eligibility definitions, a Tier 2 screening decision framework, component-specific search procedures, explicit quantitative-synthesis thresholds, document-type-specific policy appraisal methods, and more detailed procedures for qualitative synthesis and reviewer responsibilities. Two new extended-data files also provide the complete planned database strategies and the operational protocol for the policy framework analysis. The updated materials are available through the revised Zenodo record: https://doi.org/10.5281/zenodo.21825405 We again thank the reviewer for the constructive recommendations, which have helped us strengthen the clarity, transparency, and reproducibility of the protocol.

# Reviewer report: Reviewer response for version 1

**DOI:** 10.21956/openreseurope.25974.r76507

**Published:** 2026-06-30

**Authors:** Henri Tilga

**Affiliations:** 1 Institute of Sport Sciences and Physiotherapy, Faculty of Medicine, University of Tartu, Tartu, Estonia

Overall, this protocol addresses a highly relevant and timely topic by focusing on physical education within hospital school settings, an area that has received remarkably limited scientific attention despite its potential educational, physical, and psychosocial importance. The proposed integration of a systematic review, an umbrella review, and a policy framework analysis represents a novel and comprehensive methodological approach that has the potential to make a meaningful contribution to both research and practice. The manuscript is generally well organized, the rationale is convincing, and the protocol follows recognized methodological standards, including PRISMA-P and PROSPERO registration. Nevertheless, several aspects could be further strengthened to improve methodological transparency, conceptual clarity, and overall readability before the review is conducted.

 Please consider strengthening the conceptual distinction between physical education, physical activity, therapeutic exercise, rehabilitation, and play-based movement throughout the manuscript. Although these concepts are acknowledged as related, the eligibility criteria suggest that they may overlap considerably during study selection, particularly within Tier 2. A clearer operational definition of physical education in hospital school settings would reduce ambiguity and improve the reproducibility of the review process. Perhaps the authors could explicitly define the essential characteristics that distinguish educational physical education interventions from therapeutic or clinical exercise programmes while also explaining how borderline cases will be handled during screening.

 The Introduction provides an appropriate justification for the importance of physical education during hospitalization; however, several arguments regarding the physical, psychological, educational, and social benefits of physical activity are repeated throughout the section. Please consider condensing these recurring statements and directing greater attention toward the specific knowledge gap that motivates this protocol. A more concise introduction would improve readability while allowing greater emphasis on why previous reviews have been insufficient and why the proposed combination of systematic review, umbrella review, and policy analysis is necessary.

 The methodological description is generally comprehensive, although several sections would benefit from greater precision. Please consider providing separate and more detailed descriptions of the search strategies for the umbrella review, the systematic review of primary studies, and the policy framework analysis. Because these components address different research questions and involve different evidence sources, readers would benefit from a clearer explanation of how each search will be conducted and how the corresponding eligibility criteria will differ. This would further enhance the transparency and reproducibility of the protocol.

 The policy framework analysis represents one of the most innovative aspects of the protocol; however, its methodology could perhaps be described in greater detail. It would be useful to clarify which categories of policy documents will be eligible for inclusion, how grey literature searches will be standardized across countries, and how expert consultation will be documented to minimize potential selection bias. Furthermore, although AGREE II is an established instrument for evaluating clinical practice guidelines, please consider providing additional justification for its application to legislation, governmental regulations, and broader policy documents, or alternatively explain whether supplementary appraisal criteria will be used for documents that fall outside the intended scope of AGREE II.

 The planned evidence synthesis is ambitious and reflects the comprehensive nature of the review. Nevertheless, perhaps the protocol could specify more clearly the conditions under which quantitative meta-analysis will be undertaken. Considering the anticipated methodological and clinical heterogeneity across study designs, interventions, populations, and outcome measures, it appears likely that narrative synthesis will constitute the primary analytical approach. Defining minimum criteria for statistical pooling, together with identifying several priority outcome domains for quantitative synthesis and GRADE assessment, would strengthen the methodological rigor of the protocol and improve readers' expectations regarding the final review.

 The inclusion of qualitative studies is an important strength because it allows the review to capture barriers, facilitators, stakeholder experiences, and implementation issues that quantitative studies alone cannot adequately address. However, please consider expanding the description of the qualitative synthesis by providing greater detail regarding the coding process, theme development, reviewer calibration, and procedures that will be used to ensure analytical credibility and reduce interpretative subjectivity. The recently added section describing the responsible use of artificial intelligence is appropriate and reflects current methodological recommendations. Nevertheless, it may be helpful to emphasize consistently throughout the manuscript that all methodological decisions will remain under direct human responsibility and that any automated tools will serve exclusively as supportive instruments.

 The protocol generally follows accepted reporting standards, although some procedural descriptions appear repeatedly across several sections. For example, the explanation that two independent reviewers will conduct screening, data extraction, and quality assessment with disagreements resolved by discussion or consultation with a third reviewer is repeated multiple times. Please consider presenting this workflow once in a concise manner and referring back to it where appropriate. This would improve the overall flow of the manuscript without reducing methodological transparency.

Is the study design appropriate for the research question?

Yes

Are sufficient details of the methods provided to allow replication by others?

Yes

Is the rationale for, and objectives of, the study clearly described?

Yes

Are the datasets clearly presented in a useable and accessible format?

Yes

Reviewer Expertise:

Physical education

I confirm that I have read this submission and believe that I have an appropriate level of expertise to confirm that it is of an acceptable scientific standard, however I have significant reservations, as outlined above.

# Response

**Published:** 2026-08-17

**Authors:** Simone Ciaccioni

Dear Dr Tilga,

 We sincerely thank you for your positive assessment of the relevance, novelty, and methodological approach of our protocol, as well as for your detailed and constructive recommendations. Your comments helped us improve the conceptual clarity, methodological transparency, reproducibility, and readability of the manuscript. We have revised the manuscript substantially and provide our point-by-point responses below.

**Comment 1. Conceptual distinction and Tier 2 eligibility**

 Please consider strengthening the conceptual distinction between physical education, physical activity, therapeutic exercise, rehabilitation, and play-based movement throughout the manuscript. A clearer operational definition of physical education in hospital school settings would reduce ambiguity and improve reproducibility. The authors should define the essential characteristics distinguishing educational physical education interventions from therapeutic or clinical exercise programmes and explain how borderline cases will be handled.

**Response:**

 We agree and have strengthened these distinctions throughout the Introduction, eligibility criteria, and screening procedures. A new terminology and screening decision table, now presented as Table 1, operationally distinguishes physical education from physical activity, therapeutic exercise, rehabilitation, and active play. For inclusion in Tier 2, an intervention must have an explicit educational purpose, such as curriculum-related learning, development of movement competence, knowledge, participation, self-regulation, or personal and social learning through movement. In addition, the intervention must either:

1. be formally integrated into the hospital-school programme; or
2. involve educational staff in its planning, delivery, or assessment.

Therapeutic exercise, physiotherapy, rehabilitation, and active play delivered solely for clinical treatment, symptom management, or recreation will therefore be excluded from Tier 2. Such interventions may nevertheless be represented in Tier 1 when included in an eligible systematic review. For borderline cases combining educational, therapeutic, and recreational elements, the explicit educational purpose remains mandatory. These cases will undergo independent duplicate full-text assessment, and study authors will be contacted where the educational purpose or hospital-school connection cannot be determined from the report.

**Comment 2. Introduction and knowledge gap** The Introduction appropriately justifies the relevance of physical education during hospitalization, but several statements concerning the physical, psychological, educational, and social benefits of physical activity are repeated. Please consider condensing these statements and placing greater emphasis on the specific knowledge gap and the need for the combined systematic review, umbrella review, and policy analysis.

**Response:**

 We agree and have substantially condensed and reorganised the Introduction. Repeated descriptions of the general benefits of physical education and physical activity have been removed. The revised Introduction now focuses more directly on:

1. the limited and inconsistent provision of physical education in hospital schools;
2. the distinction between educational physical education and related clinical or recreational movement activities;
3. the fragmentation of evidence across education, rehabilitation, psychology, nursing, and sport sciences;
4. the lack of an integrated synthesis of empirical and policy evidence; and
5. the rationale for combining two evidence-synthesis tiers with a parallel policy framework analysis.

We have also added five explicit objectives addressing the characteristics and delivery of physical education interventions, their safety and outcomes, potentially transferable evidence from broader hospital-based physical activity interventions, relevant policy and regulatory provisions, and the integration of empirical and policy findings.

**Comment 3. Separate search strategies** Please consider providing separate and more detailed descriptions of the search strategies for the umbrella review, the systematic review of primary studies, and the policy framework analysis. Because these components address different research questions and involve different evidence sources, readers would benefit from a clearer explanation of how each search will be conducted and how the corresponding eligibility criteria will differ.

**Response:**

 We agree and have comprehensively revised the search-methods section. Separate search structures are now presented for:

- Tier 1, the umbrella review;
- Tier 2, the systematic review of primary studies; and
- the policy framework analysis.

For Tier 1, hospital-school terminology will not be required because the aim is to identify broader systematic-review evidence concerning physical activity, exercise, and movement interventions for hospitalised children and adolescents. For Tier 2, the search will prioritise sensitivity by combining hospital-school or in-hospital education concepts with broad terms relating to physical education, physical activity, exercise, movement, sport, and active play. The educational eligibility decision rule will subsequently be applied during full-text screening. The policy search will cover official international, European, national, and regional organisation websites, ministries of education and health, legal databases, curriculum authorities, Youth Wiki, and recognised hospital-school networks. Searches will use English and relevant local-language terminology. Complete line-by-line database strategies and a detailed operational protocol for the policy framework analysis have also been added as two new extended-data files. Procedures for citation searching, grey-literature identification, search updating, translation, documentation, and reporting of search deviations are now described more explicitly.

**Comment 4. Policy framework analysis**

 The policy framework analysis is innovative but requires more methodological detail. The authors should clarify eligible document categories, how grey-literature searches will be standardised across countries, how expert consultation will be documented, and whether appraisal criteria other than AGREE II will be used for documents outside the scope of clinical guidelines.

**Response:**

 We agree and have expanded the policy-analysis methods substantially. Eligible documents are now explicitly defined as binding and non-binding instruments, including:

- legislation;
- regulations;
- ministerial decrees and circulars;
- curriculum frameworks;
- national and regional strategies;
- official standards;
- implementation guidance; and
- international normative or recommendation documents.

A standardised country-search template will document the official domains searched, English and local-language terms, document categories, search dates, result counts, and reasons for exclusion. Where official website search functions are inadequate, targeted searches restricted to official domains will be used. Expert consultation will be used only to identify potentially missing documents. All experts will receive the same written request, and the search log will record their role, country, date of contact, and nominated documents. Each nominated document will be independently assessed for eligibility and source authenticity. Expert opinions themselves will not be analysed as evidence. We have also revised the appraisal strategy. AGREE II will be applied only to documents that meet the characteristics of a practice or implementation guideline. Other policy texts will be assessed using the JBI approach for textual policy evidence, while grey-literature characteristics will additionally be documented using the AACODS checklist. Laws and regulations will be characterised according to their issuing authority, legal force, currency, scope, accessibility, and implementation provisions.

**Comment 5. Quantitative synthesis and priority outcomes**

 The planned synthesis is ambitious. Please specify more clearly the conditions under which quantitative meta-analysis will be undertaken. Narrative synthesis is likely to be the primary approach. Defining minimum criteria for pooling and priority outcome domains for quantitative synthesis and GRADE assessment would strengthen the protocol.

**Response:**

 We agree and have revised the synthesis plan accordingly. Narrative synthesis is now explicitly identified as the primary analytical approach for both evidence-synthesis tiers. Meta-analysis will be considered only when at least three independent studies are sufficiently comparable in terms of:

- population;
- intervention purpose and content;
- study design;
- comparator;
- outcome construct; and
- assessment time point.

Pooling will not be undertaken where participant data would be double-counted, and randomised and non-randomised studies will not be combined in the same meta-analysis. Decisions to pool will be based primarily on clinical, educational, and methodological comparability rather than statistical heterogeneity alone. Case reports, case series, and descriptive evidence will be used for mapping and narrative interpretation rather than for pooled estimates of effectiveness. We have also organised the predefined priority domains into two complementary synthesis strands. Safety and feasibility/implementation, together with educational participation or engagement, will primarily contribute to an implementation/process synthesis drawing on quantitative descriptive, qualitative and mixed-method evidence. Physical functioning or motor competence and psychosocial well-being or health-related quality of life will be prioritised for comparative quantitative synthesis and GRADE assessment. However, this distinction is not absolute: safety or educational participation outcomes will also be eligible for quantitative synthesis and GRADE when sufficiently comparable comparative evidence is available. This structure is intended to keep the synthesis proportionate to the anticipated evidence base while avoiding the application of the same analytical approach to conceptually different types of evidence. Other clinical, cognitive, and academic outcomes will be mapped and synthesised narratively.

**Comment 6. Qualitative synthesis and artificial intelligence**

 Please expand the description of the qualitative synthesis, including coding, theme development, reviewer calibration, and procedures to reduce interpretative subjectivity. The manuscript should also consistently clarify that artificial-intelligence or automated tools will be supportive only and that all methodological decisions will remain under human responsibility.

**Response:**

 We agree and have added a more detailed qualitative synthesis procedure. Qualitative findings will be synthesised using a three-stage thematic-synthesis process comprising:

1. line-by-line coding;
2. development of descriptive themes; and
3. generation of analytical themes.

Two reviewers will independently code an initial sample representing different settings and participant perspectives. They will compare their interpretations and agree on a provisional codebook. One reviewer will then code the remaining findings, while a second reviewer will verify the coding and theme development. Analytical transparency will be supported by an audit trail documenting:

- the codebook and its revisions;
- reflexive notes;
- coding decisions;
- divergent cases; and
- changes made during theme development.

Participant quotations will also be distinguished from study-author interpretations. The section on artificial intelligence and automation has been revised to state explicitly that such tools, if used, will be limited to supportive tasks such as deduplication, record organisation, translation support, or citation prioritisation. Automated outputs will not determine study eligibility, extraction, appraisal, qualitative coding, theme development, certainty assessments, or interpretation. All outputs will be verified by human reviewers, who will retain direct responsibility for every methodological decision. The tool name, developer, version, purpose, date of use, verification procedures, and any identified errors will be documented in the final review report.

**Comment 7. Repetition of reviewer procedures**

 The description of the two-reviewer process is repeated across several sections. Please consider presenting this workflow once and referring back to it where appropriate.

**Response:**

 We agree and have consolidated these procedures in a new reviewer-workflow table, now presented as Table 3. The table summarises reviewer responsibilities and disagreement-resolution procedures for:

- search validation;
- title, abstract, and full-text screening;
- data extraction;
- risk-of-bias and critical appraisal;
- qualitative synthesis;
- certainty assessment; and
- policy-document analysis.

Repetitive descriptions in the individual methodological sections have consequently been shortened, and the sections now refer to the common workflow where appropriate. We again thank the reviewer for the careful and constructive assessment. The recommendations have helped us improve the conceptual precision, transparency, feasibility, and readability of the protocol.

# Reviewer report: Reviewer response for version 1

**DOI:** 10.21956/openreseurope.25974.r75443

**Published:** 2026-06-24

**Authors:** Federico Roggio

**Affiliations:** 1 Department of Biomedical and Biotechnological Sciences, Section of Anatomy, Histology and Movement Science, School of Medicine,, University of Catania, Catania, Italy

Thank you for the opportunity to review the manuscript entitled “MSCA HOPE – Physical education in hospital schools: A protocol paper for systematic and umbrella reviews with policy framework analysis.” The manuscript addresses a timely and underexplored topic, with potential relevance for hospital education, paediatric health, and policy development. The proposed integration of a systematic review, an umbrella review, and a policy framework analysis is ambitious and valuable.

 The manuscript is relevant and promising, but it requires clearer methodological definitions, a more reproducible search strategy, a more cautious synthesis plan, stronger detail on the policy analysis, and a more consistent protocol-oriented tone.

 Comments:

**1. The scope of the review should be better defined**

 The manuscript includes several related concepts, such as physical education, physical activity, therapeutic exercise, active play, rehabilitation, and movement-based educational activities. However, the boundaries between these categories are not always clear. This is particularly important for Tier 2, which should focus on physical education in hospital school settings. The authors should provide a clearer operational definition of what qualifies as “physical education” in this specific context. A short terminology table or a screening decision rule would help readers understand which interventions will be included or excluded.

**2. The Introduction is somewhat repetitive**

 The Introduction repeatedly states that physical education and physical activity have physical, psychosocial, educational, and developmental benefits. While this rationale is important, the repetition reduces the directness of the argument. The authors should condense these passages and focus more clearly on the specific gap addressed by this protocol: the lack of synthesised evidence and policy guidance on PE in hospital schools.

**3. The synthesis plan should be presented more cautiously**

 The protocol states that meta-analyses may be conducted if sufficient homogeneous data are available. However, Tier 2 is expected to include highly heterogeneous evidence, including RCTs, non-randomised studies, observational studies, qualitative studies, mixed-methods studies, and case reports. The authors should specify more clearly under which conditions a meta-analysis will be performed. They should also identify a limited number of key outcome domains to be prioritised for synthesis and GRADE assessment. Otherwise, the synthesis plan risks appearing too ambitious for the likely evidence base.

**4. Section 2.3 should be revised to avoid repetition and improve reproducibility**

 The section on data sources and search strategy partly repeats the information already presented in Table 1; this section should provide more operational detail.

 The authors should present separate search strategies for the umbrella review, the systematic review of primary studies, and the policy framework analysis. These components have different aims and should not rely on a single generic search structure.

**5. The policy framework analysis requires more detail**

 The policy analysis is one of the most interesting aspects of the protocol, but it is currently under-specified. The authors should clarify which types of policy documents will be eligible, and how grey literature will be searched. They should also explain how expert consultation will be documented and how potential selection bias will be minimised. In addition, the use of AGREE-II should be justified, since this tool may be appropriate for guidelines but not necessarily for laws, regulations, or ministerial documents.

**6. Qualitative evidence and expert input should be handled more transparently**

 The inclusion of qualitative studies is appropriate, especially for exploring barriers, facilitators, feasibility, and stakeholder perspectives. However, the protocol should explain more clearly how interview-based or qualitative evidence will be analysed.

 They should clarify how they will limit subjectivity in qualitative synthesis. If text-mining or AI-assisted tools are considered, these should be described as supportive tools only, with final decisions remaining under human reviewer responsibility.

**7. The tone should be more consistent with a protocol paper**

 Although the manuscript is presented as a protocol, some sections sound closer to a guidance document or position paper. In some passages, the wording seems to anticipate the conclusions or practical impact of the future review.

 The authors should revise the use of verb tenses and phrasing to distinguish more clearly between planned methods, expected outputs, and conclusions that can only be drawn after the review is completed. Expressions such as “will provide evidence” or “will support improvements” could be softened to “is intended to provide evidence” or “may inform future practice and policy”.

**8. Minor aspect:  repeated description of the two-reviewer process could be streamlined**

 The manuscript repeatedly states that two reviewers will independently perform screening, data extraction, and appraisal, with disagreements resolved by consensus or by a third reviewer. This is methodologically correct, but the repetition affects readability. The authors could summarise this workflow once, possibly in a short table, indicating the role of reviewers across screening, extraction, risk-of-bias assessment, certainty assessment, and policy document analysis.

Is the study design appropriate for the research question?

Yes

Are sufficient details of the methods provided to allow replication by others?

Partly

Is the rationale for, and objectives of, the study clearly described?

Partly

Are the datasets clearly presented in a useable and accessible format?

No

Reviewer Expertise:

Public health - human movement sciences

I confirm that I have read this submission and believe that I have an appropriate level of expertise to confirm that it is of an acceptable scientific standard, however I have significant reservations, as outlined above.

# Response

**Published:** 2026-08-17

**Authors:** Simone Ciaccioni

Dear Dr Roggio, we sincerely thank you for your careful and constructive assessment of our protocol. Your comments helped us clarify the conceptual scope of the review, strengthen the reproducibility of the methods, and adopt a more appropriately cautious protocol-oriented tone. We have substantially revised the manuscript and the associated extended data. Our point-by-point responses are provided below.

**Comment 1. The scope of the review should be better defined** 

 The manuscript includes several related concepts, such as physical education, physical activity, therapeutic exercise, active play, rehabilitation, and movement-based educational activities. However, the boundaries between these categories are not always clear. This is particularly important for Tier 2, which should focus on physical education in hospital school settings. The authors should provide a clearer operational definition of what qualifies as “physical education” in this specific context. A short terminology table or a screening decision rule would help readers understand which interventions will be included or excluded.

**Response:**

 We agree and have substantially clarified the conceptual and operational boundaries of Tier 2. First, the Introduction now distinguishes physical education from physical activity, therapeutic exercise, rehabilitation, and active play according to their primary purpose, intended outcomes, and professional responsibilities. Second, we added a new terminology and screening decision table, now presented as Table 1. For Tier 2, an intervention must meet the following mandatory criterion:

1. It must have an explicit educational purpose, such as curriculum-related learning, development of movement competence, knowledge, participation, self-regulation, or personal and social learning through movement.

In addition, at least one of the following criteria must be met:

1. The activity is formally integrated into the hospital-school programme; or
2. Educational staff are involved in its planning, delivery, or assessment.

Therapeutic exercise, physiotherapy, rehabilitation, and active play delivered solely for clinical treatment, symptom management, or recreation are therefore excluded from Tier 2. They may nevertheless be represented in Tier 1 when included in an eligible systematic review. We have also introduced a specific decision rule for borderline interventions combining educational, therapeutic, and recreational purposes. These cases will undergo duplicate full-text assessment, and study authors will be contacted where the educational purpose or hospital-school connection remains unclear.

**Comment 2. The Introduction is somewhat repetitive**

 The Introduction repeatedly states that physical education and physical activity have physical, psychosocial, educational, and developmental benefits. While this rationale is important, the repetition reduces the directness of the argument. The authors should condense these passages and focus more clearly on the specific gap addressed by this protocol: the lack of synthesised evidence and policy guidance on PE in hospital schools.

**Response:**

 We agree and have condensed and reorganised the Introduction. The revised Introduction now follows a more direct sequence:

1. the role of hospital schools and the limited provision of physical education;
2. the conceptual distinction between physical education and related movement-based activities;
3. the fragmentation of the available empirical and policy evidence; and
4. the specific objectives of the multi-component review.

Repeated descriptions of the general benefits of physical education and physical activity have been removed. The revised rationale now focuses on the absence of an integrated synthesis of empirical evidence and policy provisions concerning physical education in hospital schools. We have also added an explicit list of five objectives concerning intervention characteristics, safety and outcomes, transferable evidence from broader hospital-based physical activity interventions, policy provisions, and the integration of empirical and policy findings.

**Comment 3. The synthesis plan should be presented more cautiously**

 The protocol states that meta-analyses may be conducted if sufficient homogeneous data are available. However, Tier 2 is expected to include highly heterogeneous evidence, including RCTs, non-randomised studies, observational studies, qualitative studies, mixed-methods studies, and case reports. The authors should specify more clearly under which conditions a meta-analysis will be performed. They should also identify a limited number of key outcome domains to be prioritised for synthesis and GRADE assessment. Otherwise, the synthesis plan risks appearing too ambitious for the likely evidence base.

**Response:**

 We agree and have revised the synthesis plan to make narrative synthesis the primary approach. Meta-analysis will now be considered only when at least three independent studies are sufficiently comparable in relation to:

- population;
- intervention purpose and content;
- study design;
- comparator;
- outcome construct; and
- assessment time point.

Effect estimates will not be pooled when doing so would result in double-counting participants, and randomised and non-randomised studies will not be combined in the same meta-analysis. Decisions concerning pooling will be based primarily on clinical, educational, and methodological comparability rather than statistical heterogeneity alone. Case reports, case series, and descriptive evidence will be used for evidence mapping and narrative interpretation and will not contribute to pooled estimates of effectiveness. We have also organised the predefined priority domains into two complementary synthesis strands. Safety and feasibility/implementation, together with educational participation or engagement, will primarily contribute to an implementation/process synthesis drawing on quantitative descriptive, qualitative and mixed-method evidence. Physical functioning or motor competence and psychosocial well-being or health-related quality of life will be prioritised for comparative quantitative synthesis and GRADE assessment. However, this distinction is not absolute: safety or educational participation outcomes will also be eligible for quantitative synthesis and GRADE when sufficiently comparable comparative evidence is available. This structure is intended to keep the synthesis proportionate to the anticipated evidence base while avoiding the application of the same analytical approach to conceptually different types of evidence. Other clinical, cognitive, and academic outcomes will be mapped and synthesised narratively. GRADE-CERQual will be limited to selected qualitative findings concerning safety, acceptability, barriers, facilitators, and stakeholder experiences. Certainty ratings will not be assigned to isolated case reports, descriptive mappings, or legal obligations.

**Comment 4. Section 2.3 should be revised to avoid repetition and improve reproducibility**

 The section on data sources and search strategy partly repeats the information already presented in Table 1; this section should provide more operational detail. The authors should present separate search strategies for the umbrella review, the systematic review of primary studies, and the policy framework analysis. These components have different aims and should not rely on a single generic search structure.

**Response:**

 We agree and have comprehensively revised Section 2.3. Separate search structures are now specified for:

- Tier 1, the umbrella review;
- Tier 2, the systematic review of primary studies; and
- the policy framework analysis.

The revised Table 2 summarises the databases, sources, and search concepts for each component. For Tier 1, hospital-school terminology is not mandatory because the purpose is to identify broader systematic-review evidence concerning physical activity, exercise, and movement interventions for hospitalised children and adolescents. For Tier 2, the search prioritises sensitivity by combining hospital-school and in-hospital education concepts with broad movement-related terminology. The educational screening decision rule in Table 1 will subsequently be applied during full-text screening. For the policy analysis, searches will cover official international, European, national, and regional websites, ministries, legal databases, curriculum authorities, Youth Wiki, and recognised hospital-school networks. Search terms will be adapted to relevant local languages. Complete line-by-line database strategies and the operational procedures for the policy analysis are now available as two new extended-data files. We have also specified procedures for citation searching, grey-literature identification, translation, search logging, deduplication, search updating, and the reporting of platforms, filters, dates, and protocol deviations.

**Comment 5. The policy framework analysis requires more detail** The policy analysis is one of the most interesting aspects of the protocol, but it is currently under-specified. The authors should clarify which types of policy documents will be eligible, and how grey literature will be searched. They should also explain how expert consultation will be documented and how potential selection bias will be minimised. In addition, the use of AGREE-II should be justified, since this tool may be appropriate for guidelines but not necessarily for laws, regulations, or ministerial documents.

**Response:**

 We agree and have expanded the policy framework analysis throughout the Methods section and in a dedicated extended-data protocol. Eligible documents are now explicitly defined as binding and non-binding instruments, including:

- legislation;
- regulations;
- ministerial decrees and circulars;
- curriculum frameworks;
- national and regional strategies;
- official standards;
- implementation guidance; and
- international normative or recommendation documents.

A standardised country-level search template will record the official domains searched, English and local-language terms, document categories, search dates, result counts, and reasons for exclusion. When official website search functions are inadequate, targeted searches restricted to official domains will be used. Historical or superseded documents will be retained only when necessary to explain policy development and will be clearly identified as such. Expert consultation will be used only as a supplementary document-identification method. All experts will receive the same written request, and the search log will document their role, country, date of contact, and nominated documents. Each nominated document will undergo the same independent eligibility assessment and source-authentication procedures as documents identified through the standardised searches. Expert opinions will not be analysed as data. We have also revised the appraisal strategy. AGREE II will now be used only for documents that meet the characteristics of a practice or implementation guideline. Other policy texts will be assessed using the JBI approach for textual policy evidence, supplemented by AACODS criteria for grey-literature sources. Laws and regulations will be characterised according to issuing authority, legal force, currency, scope, accessibility, and implementation provisions. These differentiated procedures are intended to avoid applying a single appraisal instrument to fundamentally different types of policy evidence.

**Comment 6. Qualitative evidence and expert input should be handled more transparently**

 The inclusion of qualitative studies is appropriate, especially for exploring barriers, facilitators, feasibility, and stakeholder perspectives. However, the protocol should explain more clearly how interview-based or qualitative evidence will be analysed. They should clarify how they will limit subjectivity in qualitative synthesis. If text-mining or AI-assisted tools are considered, these should be described as supportive tools only, with final decisions remaining under human reviewer responsibility.

**Response:**

 We agree and have added a detailed qualitative synthesis procedure. Qualitative findings will be analysed using the three-stage thematic-synthesis approach:

1. line-by-line coding;
2. development of descriptive themes; and
3. generation of analytical themes.

Two reviewers will independently code an initial sample selected to represent different settings and participant perspectives. They will compare their interpretations and agree on a provisional codebook. One reviewer will then code the remaining findings, while a second reviewer will verify the coding and development of themes. A documented audit trail will retain:

- the codebook and its revisions;
- reflexive notes;
- coding decisions;
- divergent or disconfirming cases; and
- changes made during theme development.

Participant quotations will also be distinguished from the interpretations provided by study authors. We added a separate section on artificial intelligence and automation. Any such tools, if used, will be restricted to supportive tasks such as deduplication, record organisation, translation support, or citation prioritisation. Automated outputs will not determine eligibility, extraction, appraisal, qualitative coding, theme development, certainty ratings, or interpretation. All outputs will be verified by human reviewers, who will retain responsibility for every methodological decision. The tool, version, purpose, date of use, verification procedures, and identified errors will be reported. As clarified in response to Comment 5, expert consultation will identify potentially missing policy documents only. Expert views will not be treated as qualitative evidence or included in the synthesis.

**Comment 7. The tone should be more consistent with a protocol paper**

 Although the manuscript is presented as a protocol, some sections sound closer to a guidance document or position paper. In some passages, the wording seems to anticipate the conclusions or practical impact of the future review. The authors should revise the use of verb tenses and phrasing to distinguish more clearly between planned methods, expected outputs, and conclusions that can only be drawn after the review is completed. Expressions such as “will provide evidence” or “will support improvements” could be softened to “is intended to provide evidence” or “may inform future practice and policy”.

**Response:**

 We agree and have revised the tone throughout the Abstract, Introduction, Discussion, conclusion, and dissemination sections. Statements that previously appeared to anticipate findings or implementation effects have been replaced with more cautious formulations. For example, the revised manuscript states that the reviews are “intended to clarify” the evidence and policy landscape and “may inform” future stakeholder consultation, research priorities, and guideline development. The Discussion now explicitly recognises that the evidence may be sparse, heterogeneous, and at risk of bias and that the completed reviews will not, by themselves, establish universal implementation standards. It also distinguishes planned methods from possible future applications and avoids assuming that evidence from clinical physical activity interventions will be directly transferable to physical education in hospital schools.

**Comment 8. Minor aspect: repeated description of the two-reviewer process could be streamlined**

 The manuscript repeatedly states that two reviewers will independently perform screening, data extraction, and appraisal, with disagreements resolved by consensus or by a third reviewer. This is methodologically correct, but the repetition affects readability. The authors could summarise this workflow once, possibly in a short table, indicating the role of reviewers across screening, extraction, risk-of-bias assessment, certainty assessment, and policy document analysis.

**Response:**

 We agree and have consolidated the reviewer procedures in a new reviewer-workflow table, now presented as Table 3. The table summarises reviewer roles and disagreement-resolution procedures for:

- search validation;
- title, abstract, and full-text screening;
- data extraction;
- risk-of-bias and critical appraisal;
- qualitative synthesis;
- certainty assessment; and
- policy-document analysis.

The repeated descriptions in the individual methodological sections have consequently been shortened, with those sections now referring to the common workflow where appropriate.

**Responses to the assessment questions**

**Is the rationale for, and objectives of, the study clearly described?** **Original assessment: Partly** **Response:**

 The Introduction has been condensed and refocused on the specific empirical and policy gap addressed by the protocol. The revised Abstract now states the overall aim explicitly, and the Introduction presents five clearly defined objectives covering Tier 1, Tier 2, the policy analysis, and the planned integration of findings.

**Are sufficient details of the methods provided to allow replication by others?** **Original assessment: Partly** **Response:**

 We have added component-specific eligibility criteria, operational definitions, a Tier 2 screening decision rule, separate search frameworks, predefined synthesis thresholds, detailed qualitative procedures, policy-document selection and appraisal methods, and a consolidated reviewer workflow. In addition, two new extended-data files provide the complete planned database search strategies and the operational protocol for the policy framework analysis. The Zenodo record has been updated accordingly.

**Are the datasets clearly presented in a usable and accessible format?** **Original assessment: No** **Response:**

 As this article is a protocol, no empirical study dataset has yet been generated. However, the methodological extended data supporting implementation and reproducibility of the protocol are openly available through the updated Zenodo record: https://doi.org/10.5281/zenodo.21825405 The revised Data Availability statement identifies these materials and specifies that they are available under a CC0 public-domain dedication.

 We again thank the reviewer for the constructive recommendations, which have materially improved the clarity, transparency, and feasibility of the protocol.
